# Robust Brain-Machine Interface Design Using Optimal Feedback Control Modeling and Adaptive Point Process Filtering

**DOI:** 10.1371/journal.pcbi.1004730

**Published:** 2016-04-01

**Authors:** Maryam M. Shanechi, Amy L. Orsborn, Jose M. Carmena

**Affiliations:** 1 Department of Electrical Engineering, Viterbi School of Engineering, University of Southern California, Los Angeles, California, United States of America; 2 Department of Electrical Engineering and Computer Sciences, University of California, Berkeley, Berkeley, California, United States of America; 3 University of California, Berkeley—University of California, San Francisco Graduate Group in Bioengineering, San Francisco, California, United States of America; 4 Helen Willis Neuroscience Institute, University of California, Berkeley, Berkeley, California, United States of America; Indiana University, UNITED STATES

## Abstract

Much progress has been made in brain-machine interfaces (BMI) using decoders such as Kalman filters and finding their parameters with closed-loop decoder adaptation (CLDA). However, current decoders do not model the spikes directly, and hence may limit the processing time-scale of BMI control and adaptation. Moreover, while specialized CLDA techniques for intention estimation and assisted training exist, a unified and systematic CLDA framework that generalizes across different setups is lacking. Here we develop a novel closed-loop BMI training architecture that allows for processing, control, and adaptation using spike events, enables robust control and extends to various tasks. Moreover, we develop a unified control-theoretic CLDA framework within which intention estimation, assisted training, and adaptation are performed. The architecture incorporates an infinite-horizon optimal feedback-control (OFC) model of the brain’s behavior in closed-loop BMI control, and a point process model of spikes. The OFC model infers the user’s motor intention during CLDA—a process termed intention estimation. OFC is also used to design an autonomous and dynamic assisted training technique. The point process model allows for neural processing, control and decoder adaptation with every spike event and at a faster time-scale than current decoders; it also enables dynamic spike-event-based parameter adaptation unlike current CLDA methods that use batch-based adaptation on much slower adaptation time-scales. We conducted closed-loop experiments in a non-human primate over tens of days to dissociate the effects of these novel CLDA components. The OFC intention estimation improved BMI performance compared with current intention estimation techniques. OFC assisted training allowed the subject to consistently achieve proficient control. Spike-event-based adaptation resulted in faster and more consistent performance convergence compared with batch-based methods, and was robust to parameter initialization. Finally, the architecture extended control to tasks beyond those used for CLDA training. These results have significant implications towards the development of clinically-viable neuroprosthetics.

## Author Summary

Brain-machine interfaces (BMI) decode subjects’ intended movements from neural activity. Despite significant advances, performance, robustness, and extendibility remain key challenges. Current BMIs use decoders such as Kalman filters whose parameters are estimated in closed-loop BMI operation. However, current decoders do not model the spikes directly and hence may limit the time-scale of neural processing, control, and parameter estimation. Here we develop a novel BMI training architecture for spike-event-based control and parameter estimation, and show that it enables robust control and extends to various tasks. Moreover, we propose a control-theoretic framework for closed-loop decoder training. The BMI incorporates an optimal feedback-control (OFC) model of brain’s control behavior to infer its motor intention. The BMI uses a point process to model the spikes; this enables the subject to control the movement and the decoder to estimate the parameters using spike events. Using closed-loop experiments in a non-human primate, we show that the OFC model improves performance compared with current intention estimation techniques; that spike-event-based adaptation enables faster performance convergence compared with current batch-based methods, and is robust to parameter initialization; and that the architecture extends to various tasks. This architecture has significant implications for future clinically-viable BMIs.

## Introduction

**Fig 1:**
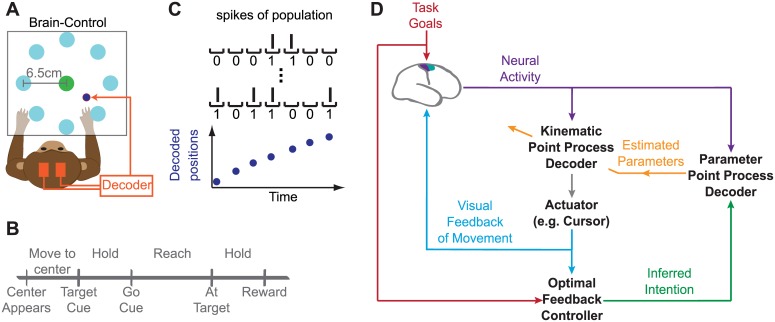
Adaptive OFC-PPF BMI architecture. (A) Monkey performing the self-paced delayed center-out movement task in brain control. The subject’s arms were confined within a primate chair in brain control. (B) Timeline of the center-out task (see Experimental Procedures for details). (C) Adaptive OFC-PPF converts the spiking activity into a discrete time-series of 0’s and 1’s by binning the spikes in small intervals containing at most one spike; this binary time-series is modeled as a point process. We thus perform the decoding and parameter adaptation with every binary spike event. (D) Adaptive OFC-PPF architecture. The architecture models the brain in closed-loop BMI control as an infinite-horizon optimal feedback-controller to infer its intended velocity during adaptation. The inputs to the infinite-horizon optimal feedback-controller model are the visual feedback of the decoded cursor kinematics (that the monkey observes) and the instructed target position (i.e., task goal). The inferred intended velocity is input to a point process filter for each neuron, which estimates the neuron’s parameters with every 0 and 1 spike event. These estimated parameters are used in the kinematic PPF decoder that decodes the kinematics with every 0 and 1 spike event. Initially, the architecture can provide assisted training to the subject by decoding the kinematics using a target-directed PPF decoder (see Methods). After assisted training is complete, a random-walk PPF is used to decode the kinematics. Once performance converges, adaptation stops and the trained random-walk PPF is used by the monkey to perform various BMI tasks, such as the center-out or the target-jump tasks.

Brain-machine interfaces (BMI) have the potential to enable motor function in individuals with neurological injury or disease [1–5]. BMI research has demonstrated that human and non-human primates can use their neural activity to directly control computer cursors or robotic arms (e.g., [6–28]). BMIs record the neural activity from motor cortical areas, use a mathematical transform termed the “decoder” to convert this activity into control commands for an external device, and provide visual feedback of the generated movement to the subject (Fig 1A). Various decoders such as linear regression, population vector, and Kalman filters (KF) have been used in real-time BMIs [29]. Once a decoding model is selected, its parameters need to be estimated for each subject. These parameters are often found in open loop in a training session by solving for the parameter values that best describe the neural activity in response to executed or imagined movement. However, the relationship between neural activity and movements can be different for movements of a natural arm versus a BMI cursor, and the decoder needs to be adjusted to better handle the latter relationship[6, 8, 15, 30]. Hence methods that fit the decoder parameters in closed-loop BMI operation [6, 11, 17, 19–21, 23, 31], referred to as closed-loop decoder adaptation (CLDA), can improve BMI performance [20, 21].

Recent works have used KF decoders and combined them with a specific form of CLDA to achieve proficient BMI control [20, 21]. Typical decoders such as the KF take as input the spike counts in bins, typically of 50–100 ms width, and assume a Gaussian distribution on these counts. Hence using a KF decoder, users can control the BMI once per bin (i.e., once every 50–100 ms). The CLDA process, in turn, consists of three steps: (1) a method to initialize the decoder, (2) a method to estimate the user’s velocity intention during CLDA, which is referred to as intention estimation, and (3) an algorithm to fit the decoder parameters based on the estimated intentions and the recorded neural activity. To perform CLDA, decoder parameters are first initialized based on arm reaching movements [20], visual feedback of cursor movements (referred to as visual feedback seed; see Methods) [21], or even arbitrarily [21]. The initialized decoder is then used by the subject to make brain-controlled movements towards visual targets on the computer screen (Fig 1A and 1B). To perform intention estimation, current methods referred to as CursorGoal find the intended velocity at each time by rotating the cursor’s decoded velocity vector towards the target while keeping its magnitude (i.e., speed) unchanged, and by equating it to zero when at the target [20]. Decoder parameters are then refit by collecting batches of neural activity on the time-scale of minutes and inferring the subject’s intended velocity in these batches. In each batch, a new set of parameters are fit using maximum-likelihood techniques[20, 21, 31]. Parameter estimates from previous batches are then either replaced with these new estimates [20] or are smoothly changed either continuously [31], or intermittently (e.g., once every 90 sec) using a technique termed SmoothBatch [21], which converges to a good solution even when not initialized based on arm movements.

Here we develop a novel closed-loop BMI training architecture termed adaptive optimal feedback-controlled (OFC) point process filter (PPF) (Fig 1C and 1D) to achieve two main goals; (1) to build a closed-loop BMI architecture that enables the subject to control the BMI at the time-scale of the spike events—defined as the presence or absence of a spike at a given time—, and that allows the decoder parameters to be adapted dynamically at this time-scale and with every spike event (see S1 Text for a summary of time-scales involved in CLDA-based BMIs); (2) to provide a unified and general control-theoretic CLDA framework within which intention estimation, assisted training, and adaptation can be performed, in contrast to current approaches.

First, while CLDA-based decoders such as the KF have yielded good performance, these decoders constrain the time-scale at which the spikes can be processed and hence the time-scale at which the subject can control the BMI based on the bin-width used for spike count calculation (typically once every 50–100ms [14, 19–21, 28, 32]; see also S1 Text). Moreover, current batch-based CLDA techniques typically update the decoder parameters at slow adaptation time-scales. However, time-scales of control and adaptation may play an important role in ultimate BMI performance. Therefore designing decoders that are not limited to a particular time-scale because of modeling assumptions (S1 Text) would provide a powerful tool to investigate the effect of the time-scales of control and adaptation. Here we develop a novel CLDA-based PPF that exhibits this property and use it to test the possibility that faster spike-event-based adaptation time-scales will improve BMI adaptation. The PPF can run at any time-scale by modeling the spikes as a series of random 0 and 1 events occurring in time [33–35]. Hence it can incorporate any time-scale down to the time-scale of the spikes (5ms here).

Second, to perform CLDA, previous approaches have successfully used various ad-hoc methods for assisted training and intention estimation. However, a unified and systematic framework that can be used to perform all these manipulations does not exist. For example, some studies do not incorporate assisted training during CLDA [20, 21] since the initial decoder is good enough to keep the subject engaged in the task. Instead, these studies rotate the decoded velocity vector to estimate intention. In other studies, assisted training has been used to help subjects reach targets with a poor initial decoder and to estimate intention (e.g., [6, 11, 36]). These studies perform assisted training by either adding to the decoded velocity vector an assistive vector that directs straight towards the target, or subtracting from the decoded velocity vector a vector perpendicular to the straight line to the target, thus attenuating the deviation. These methods often continuously or manually change the ratio of the assistive vector to the decoded vector in the sum to adjust how much assistance is provided to the subject. Some studies have used a combination of these methods (e.g.,[36]). These ad-hoc approaches can make convergence trainer-dependent and significantly reduce robustness. Highly variable levels of assistance can also complicate the interpretation of performance during adaptation. Finally, the existing CursorGoal method of velocity intention estimation is based on an assumption of straight reaches towards the target [20] and does not infer a speed intention; instead it sets the intended speed equal to the decoded speed, which may not be accurate during CLDA as decoder parameters are still being estimated. Here we propose a general unified control-theoretic framework within which full intention estimation (e.g., both direction and speed of velocity intention), assisted training, and adaptation can be performed across diverse BMI setups (see also Discussion).

Motivated by the above, we develop the novel closed-loop adaptive OFC-PPF (Fig 1C and 1D) using two main components: a point process model of the spikes and an infinite-horizon optimal feedback control model of the brain’s control behavior in the BMI paradigm. In our previous open-loop work [26, 37–39], we solved the problem of joint decoding of target and trajectory for the special case of straight target-directed movements and when decoder parameters were assumed to be known and found in open loop. Here we construct the first closed-loop PPF BMI training architecture, which fits the decoder parameters robustly and in closed loop, results in a decoder that can control various tasks and is not specific to straight target-directed movements, and allows for spike-event-based BMI control and adaptation.

To achieve our first goal, adaptive OFC-PPF models the spikes as a discrete time-series of 0’s and 1’s by binning them in small intervals containing at most one spike. This process creates a time-series of spike events; the spike events indicate the presence or absence of a spike in each small interval and hence take values 1 or 0. This enables subjects to control the BMI with every 0 or 1 spike event (every 5ms here; Fig 1C); moreover, it allows the BMI to dynamically update the decoder parameters with every spike event in contrast to batch-based maximum-likelihood methods that do so on a much larger time-scale (e.g., minutes). We use the architecture to investigate whether faster spike-event-based time-scales of adaptation improve decoder convergence.

To achieve our second goal, adaptive OFC-PPF models the brain in closed-loop BMI control as an infinite-horizon optimal feedback-controller to infer velocity intention (both direction and speed) during adaptation; this is in contrast to the existing method of intention estimation (i.e., CursorGoal), which is based on the assumption of straight reaches towards a target and does not infer speed intention [20]. The OFC method uses the target and error information to estimate the monkey’s intended velocity command in reaching the target; this estimated intended velocity is consequently used to train the decoder. In sum, the OFC model not only provides a general framework for intention estimation, but also allows for speed intention estimation that is not possible in CursorGoal. The OFC model is also used to devise a new assisted training paradigm, which is automated and dynamic and enables the computation of a chance level assisted performance. Thus the infinite-horizon OFC model results in a unified framework for these manipulations. This CLDA framework may also generalize to various BMI setups by simply quantifying the corresponding prosthetic’s dynamics and task goals within the OFC model. Finally, the proposed architecture is designed to be fully modular. We use the architecture to investigate the effect of OFC intention estimation and assisted training on BMI adaptation and performance.

We evaluated the architecture in closed-loop BMI experiments in a rhesus monkey over 89 days. We ran multiple different experiments to dissociate the effect of the novel CLDA architecture components (i.e., the effect of spike-event-based adaptation, OFC intention estimation, and OFC assisted training). We find that spike-event-based adaptation results in faster performance convergence compared to batch-based methods. Moreover, this parameter convergence is independent of initialization. Our data also shows that using the OFC intention estimation in place of the existing methods of intention estimation [20] results in a PPF decoder with higher performance, suggesting that the former better approximates the user’s strategy. Moreover, the new OFC-based assisted training technique results in consistent parameter convergence across sessions and allows for a systematic way to change the level of assistance. We also find that the architecture extends control to tasks beyond those used for CLDA training, in particular a target-jump task and a target-to-target task. Taken together, these data show that adaptive OFC-PPF provides a unified framework for CLDA, and results in robust spike-event-based BMI adaptation and control that extends to various tasks.

## Materials and Methods

### Ethics Statement

All procedures were conducted in compliance with the National Institutes of Health Guide for the Care and Use of Laboratory Animals and were approved by the University of California, Berkeley Institutional Animal Care and Use Committee.

### Adaptive OFC-PPF BMI Architecture

Here we derive the adaptive OFC-PPF BMI architecture (Fig 1D). The architecture consists of two main components, a point process model of the spikes and an optimal feedback control model of the brain behavior in BMI control. In what follows, we first develop these two components and then show how to combine them to build the architecture, which consists of a point process decoder for the kinematics and decoders for each neuron’s parameters. For readers who are mainly interested in final expressions, the kinematics decoder is given in Eqs (14)–(17) and the parameter decoder for each neuron is given in Eqs (9)–(12).

#### Infinite-horizon OFC model of the brain’s control behavior in BMI

A BMI system can be modeled as an optimal feedback-control system [26, 37–39]. Similar to natural sensorimotor control [40–42], when controlling a BMI, the brain (controller) selects the next neural command based on the feedback (e.g., visual) of the current state of the prosthetic device (e.g., cursor) and the task goal. For example, when moving a cursor on the computer screen towards a visual target (Fig 1A and 1B), the goal is to reach the target and stop there. We thus model the brain in closed-loop BMI control as an optimal feedback-controller and use this model to predict the brain’s control commands during CLDA. This model can be constructed by defining an approximate forward dynamics model, quantifying the task goals as cost functions, and modeling the visual feedback. The BMI architecture uses this OFC model of the brain’s control behavior to infer the subject’s intended kinematics and to adaptively update the decoder parameter estimates.

We denote the sequence of kinematic states by **x**_0_, ⋯ ,**x**_*t*_ and assume that they evolve according to the linear dynamical model

$$\begin{array}{c} x_{t}=Ax_{t-1}+Bu_{t-1}+w_{t-1}. \end{array}$$ (1)

This is the forward dynamics model with parameters **A** and **B** that we need to select appropriately, e.g., by fitting them based on the subject’s manual movements. Here **u**_*t*_ is the brain’s control command at time *t* and **w**_*t*_ is a zero-mean white Gaussian state noise with covariance matrix **W**, which represents the uncertainty in the forward model (in prior BMI work with Kalman filters for example, the prior model did not include the **Bu**_*t*−1_ term). We denote the decoded kinematics at time *t*, which is displayed to the subject, by **x**_*t*|*t*_. We assume that the subject perfectly observes **x**_*t*|*t*_, i.e., that the visual feedback is noiseless and instantaneous. We also implicitly assume that the brain has obtained an internal forward model of the dynamics of movement in response to control commands **u**_*t*_ in the task. Evidence for formation of such internal models has been presented in prior studies using motor control tasks [42] and more recently using BMI tasks [43].

To predict the brain’s intended control command and consequently the intended kinematics, we formulate a cost function that quantifies the goal of the task and then minimize its expected value over choices of **u**_*t*_. Target-directed movement trajectories during proficient control are dependent on the desired movement duration. Hence in our work on combined decoding of target and trajectory in target-directed movements and when decoder parameters were known [26, 37, 39], we formed a finite-horizon cost function; we then decoded the trajectory by jointly estimating the movement kinematics and its horizon (i.e., duration) from neural activity. In a CLDA technique, however, parameter estimates are poor initially and hence the decoder cannot estimate the intended kinematics accurately. Consequently, we cannot estimate the intended horizon from neural activity. Moreover, targets may not be reached during the initial trials because of poor control. Hence to develop a model of the brain’s control behavior that can be used as an intention estimator during CLDA, instead of constructing a finite-horizon cost function and decoding the horizon, we form an infinite-horizon cost function. This choice is also motivated by recent motor control studies that have suggested infinite-horizon models [44–46] as a possible alternative to the finite-horizon ones [40, 41].

In our experiments, we take the state to be **x**_*t*_ = [**d**_*t*_,**v**_*t*_]^*T*^, where the components represent the cursor’s position and velocity in the two dimensions. Denoting the target position by d*, we form the cost function as

$$\begin{array}{c} J=\sum_{t=1}^{\infty }∥d_{t}-d^{*}{∥}^{2}+w_{v}∥v_{t}{∥}^{2}+w_{r}∥u_{t}{∥}^{2}, \end{array}$$ (2)

where the three terms in the sum enforce positional accuracy, stopping condition, and energetic efficiency, respectively. The stopping condition is included in the cost function since the subject is required to stop at the target for 250ms. Hence a movement that just passes through the target without stopping at the target does not result in a reward. The efficiency cost puts a penalty on large velocity commands. The weights *w*_*r*_ and *w*_*v*_ can be first empirically approximated such that the OFC model produces trajectories similar to the monkey’s natural trajectories. These weights can be then validated and refined experimentally as shown in the Results section. Given the linear Gaussian state-space model in Eq (1) and the quadratic cost function in Eq (2), the optimal control solution **u**_*t*_ at each time that minimizes the expected cost is given by a linear function of the controller’s (brain’s) estimate of the state at that time [47]. This is the standard linear-quadratic-Gaussian (LQG) solution. Given the assumption of noiseless visual feedback, the controller’s (brain’s) estimate of the state at each time is equal to the actual displayed state on the screen, **x**_*t*|*t*_, and hence OFC-PPF solves for the intended control as

$$\begin{array}{c} u_{t}=-L\left(x_{t|t}-x^{*}\right), \end{array}$$ (3)

where x* = [d*,**0**]^*T*^ is the target state for position and velocity, and **L** is the steady-state solution to the discrete form of the algebraic Riccati equation found recursively and offline [47]. This optimal feedback control policy can be interpreted as the subject’s corrective control command in response to the deviation of the visual feedback of the current prosthetic state from the target state.

#### Point process model of the spikes

Adaptive OFC-PPF uses a point process observation model of the spiking activity in closed loop. To do so, it bins the spikes in small intervals Δ that contain at most one spike (taken to be 5ms here). This generates a discrete time-series of 0’s and 1’s (Fig 1C). This discrete time-series is modeled as a point process [33–35]. The point process model enables the brain to control the BMI with every binary spike event and at a much faster time-scale compared to the commonly-used closed-loop BMI decoders, e.g., the Wiener filter or the Kalman filter. While it is possible to fit the point process parameters by designing a batch-based technique that runs on the time-scale of minutes (using the generalized-linear models (GLM) maximum likelihood methods), this would result in a slow rate of update for the parameters. Hence we use the point process modeling framework to design an algorithm that adapts the decoder parameters with every binary spike event and at a much faster time-scale compared to batch-based techniques that are often used in BMI decoders. We will demonstrate in the Results section that this spike-event-based adaptation time-scale results in much faster convergence compared to the slower time-scale of existing batch-based methods.

We denote the neural observations of the ensemble of *C* neurons by **N**_1_, ⋯ ,**N**_*t*_ where $N_{t}=\left(N_{t}^{1},\ldots ,N_{t}^{C}\right)$ is the binary spike events of the *C* neurons at time *t*. We assume that neurons’ activities are conditionally independent given the intended kinematics. Hence the point process observation model (similar to an inhomogeneous Poisson process) for the ensemble is given by [33–35]

$$\begin{array}{c} p(N_{t}|x_{t})=\prod_{c}{\left(\lambda_{c}(t|x_{t},\phi_{t}^{c})\Delta \right)}^{N_{t}^{c}}e^{-\lambda_{c}(t|x_{t},\phi_{t}^{c})\Delta } \end{array}$$ (4)

Here Δ is the time interval used to bin the spikes, taken to be small enough to contain at most one spike, $\lambda_{c}\left(t|x_{t},\phi_{t}^{c}\right)$ is the instantaneous firing rate of neuron *c* at time *t*, and $\phi_{t}^{c}$ is the model parameters for neuron *c* that need to be estimated via CLDA (see below). Motivated by cosine tuning models of the motor cortex [35, 48, 49], we take $\lambda_{c}\left(t|x_{t},\phi_{t}^{c}\right)$ for each neuron as a log-linear function of velocity in the two dimensions

$$\begin{array}{cc} \lambda_{c}\left(t|x_{t},\phi_{t}^{c}\right) & =\lambda_{c}\left(t|v_{t},\phi_{t}^{c}\right)=\exp\left(\beta_{t}^{c}+{\alpha_{t}^{c}}^{T}v_{t}\right)=\exp\left(\left[1,v_{t}^{T}\right]\phi_{t}^{c}\right), \end{array}$$ (5)

where $\phi_{t}^{c}=\left[\beta_{t}^{c};\alpha_{t}^{c}\right]$ is the parameter in this model. Note that this model can equivalently be written as

$$\begin{array}{c} \lambda_{c}\left(t|v_{t},\phi_{t}^{c}\right)=\exp(\beta_{t}^{c}+\left|\right|\alpha_{t}^{c}\left||\;\;|\right|v_{t}||\cos\left(\theta_{t}-\theta_{p}^{c}\left(t\right)\right)) \end{array}$$ (6)

where *θ*_*t*_ is the movement direction at time *t*, $\theta_{p}^{c}\left(t\right)$ is the preferred direction and $\left|\right|\alpha_{t}^{c}\left|\right|$ is the modulation depth.

#### Spike-event-based adaptation using OFC-PPF

We now combine the OFC and PPF models to develop the adaptive OFC-PPF architecture. The architecture consists of one recursive Bayesian decoder for the kinematics and one decoder for each neuron’s parameters.

To estimate the parameters, we need to accurately infer the intended velocity and solve for values that best describe the observed neural activity in response to this intention under the observation model (4). Initially, however, the output of the kinematic decoder is not an accurate estimate of the intended velocity because of poor decoder parameter estimates. Hence during the adaptation process and to estimate the parameters, we use the infinite-horizon OFC model of the brain to infer its velocity intention. All this model needs is knowledge of the task goal and of the visual feedback to the subject, which are both independent of the quality of the kinematic decoder. The visual feedback is just the decoded kinematics **x**_*t*|*t*_ and the task goal is quantified as in Eq (2). Hence the intended kinematics at each time, denoted by ${\tilde{x}}_{t}={\left[{\tilde{d}}_{t};{\tilde{v}}_{t}\right]}^{T}$, are found from Eqs (3) and (1) as

$$\begin{array}{c} {\tilde{x}}_{t}=\left(A-BL\right)x_{t|t}+BLx^{*}. \end{array}$$ (7)

The weights in the cost function (2) can be approximately chosen such that the OFC model (7) reaches the target with a movement duration similar to the monkey’s natural movement duration. We can also experimentally test the choice of the weights in the cost function as discussed in the Results section. We experimentally tested two different types of cost functions with different assumptions about neural mechanisms for control. These findings were used to select the BMI cost function.

Given the inferred velocity intention Eq (7), we need to fit the parameters of the point process model. One way to fit these would be to use batch-based methods [20, 21], such as SmoothBatch, to find the parameters of the point process model using GLM maximum-likelihood techniques [35]. For example, we can design a SmoothBatch algorithm that finds the maximum-likelihood estimate of the point process parameters in Eq (5) in batches of 90 sec length using GLM methods, and average these batch estimates over time with a half-life of 180 sec [21]. However, this approach would only update the decoder parameters and improve the decoder quality on the time-scale of minutes, which is the batch length. To allow the BMI to update the decoder parameters with every spike event, we instead develop an adaptive algorithm by building a recursive Bayesian decoder for the parameters.

A recursive Bayesian decoder consists of a prior model on the unknown states—i.e., the tuning parameters for each neuron here—and an observation model relating the neural activity to these states. The observation model is given by Eq (4). We construct the prior model for the parameters of each neuron as a random-walk state-space model given by

$$\begin{array}{c} \phi_{t}^{c}=\phi_{t-1}^{c}+q_{t-1}, \end{array}$$ (8)

where **q**_*t*_ is white Gaussian noise with covariance matrix **Q**. We used the random-walk prior model since it provides us with a dynamic model with minimal assumption and with adequate uncertainty. The only assumption of a random-walk model is continuity in the evolution of parameters. Moreover, the random-walk model incorporates adequate uncertainty (e.g., to account for model mismatch) through its stochastic noise term **q**_*t*_.

The choice of **Q** dictates the learning rate of the adaptive decoder [50]. The development of a principled algorithm for selection of the optimal learning rate or **Q** is the topic of our future work [50]; this selection can be guided by the approximate lower and upper bounds on parameter values given by neurophysiological and physical constraints, and refined using experimental tuning.

Given the prior and the observation models, we can write the recursions of the point process decoder for the parameters, which consist of a prediction step and an update step [34, 39]. Let’s denote the one step prediction mean by $\phi_{t|t-1}^{c}=E\left(\phi_{t}^{c}|N_{1:t-1}\right)$, the prediction covariance by $\Lambda_{\phi_{t|t-1}^{c}}$, the minimum mean-squared error (MMSE) estimate by $\phi_{t|t}^{c}$, and its covariance by $\Lambda_{\phi_{t|t}^{c}}$. From Eq (8), the prediction step of OFC-PPF for the parameters is found as

$$\begin{array}{cc} \phi_{t|t-1}^{c} & =\phi_{t-1|t-1}^{c} \end{array}$$ (9)

$$\begin{array}{cc} \Lambda_{\phi_{t|t-1}^{c}} & =\Lambda_{\phi_{t-1|t-1}^{c}}+Q \end{array}$$ (10)

Since the observation model is a point process, to find the update step we make a Gaussian approximation to the posterior [34]. We have provided the recursions of a general PPF in S2 Text and shown how the parameter decoder can be obtained from these recursions given the log-linear rate function in Eq (5). The update step for the instantaneous firing rate function in Eq (5) can be found as

$$\begin{array}{cc} {\Lambda_{\phi_{t|t}^{c}}}^{-1} & ={\Lambda_{\phi_{t|t-1}^{c}}}^{-1}+s_{t}s_{t}^{T}\lambda_{c}\left(t|{\tilde{v}}_{t},\phi_{t|t-1}^{c}\right)\Delta \end{array}$$ (11)

$$\begin{array}{cc} \phi_{t|t}^{c} & =\phi_{t|t-1}^{c}+\Lambda_{\phi_{t|t}^{c}}s_{t}\left(N_{t}^{c}-\lambda_{c}\left(t|{\tilde{v}}_{t},\phi_{t|t-1}^{c}\right)\Delta \right) \end{array}$$ (12)

where $s_{t}={\left[1,{\tilde{v}}_{t}^{T}\right]}^{T}$ (see Eq (5)), and ${\tilde{v}}_{t}$ (i.e., the intended velocity) is given as in Eq (7). Hence adaptive OFC-PPF estimates each neuron’s parameters at each time step using Eqs (9)–(12).

Intuitively, in the prediction step Eqs (9) and (10) we use the random-walk state model in Eq (8) to move the parameter estimate forward in time. Since the random-walk model enforces continuity, the predicted parameters in Eq (9) are the same as the previous estimated parameters; but the prediction covariance in Eq (10) is larger to account for uncertainty and model-mismatch. In the update step Eqs (11) and (12), the estimate is found by making a correction or update to the prediction. To do so, we compare the actual spike observation at time *t*, $N_{t}^{c}$ (whether 1 or 0), with the predicted probability of having a spike at time *t*, $\lambda_{c}\left(t|{\tilde{v}}_{t},\phi_{t|t-1}^{c}\right)\Delta$ (predicted because we use the predicted parameter $\phi_{t|t-1}^{c}$ to compute this probability). Note that this predicted probability is also equivalent to the predicted expected number of spikes at time *t*, as we have a Bernoulli random variable per bin. Hence the correction is (1- predicted probability of a spike)—or equivalently (1-predicted expected value of the number of spikes)—if a spike occurs and (0- predicted probability of a spike)—or equivalently (0-predicted expected value of the number of spikes)—if no spike occurs. If a spike occurs and the predicted probability of a spike is high, this correction is small (since our predicted parameter was probably close to the true value) and vice versa. Therefore the estimate is a combination of the prediction and the correction terms (see also S3 Text).

It is important to note that adaptive OFC-PPF does not perform joint estimation of parameters and kinematics as is done in the simulation studies in [34, 51]. Instead, given the poor initial parameter estimates and hence the poor initial kinematic decoder, we rely on the OFC model to provide the intended kinematics to the parameter estimator as in Eq (7). This ensures that the poor decoded kinematics do not disrupt the adaptation of the parameters. The disruption in parameter convergence can happen as a result of joint estimation even if a goal-directed prior kinematic model is used to guide the decoded kinematics towards the target (for example the OFC goal-directed model in our assisted training paradigm; see below). This is because, unlike our approach, joint estimation requires a prior joint distribution to be placed on the kinematics and parameters. While this prior joint distribution can be pre-selected in a simulation study, it cannot be easily defined in actual BMI experiments as parameters and their uncertainty are initially unknown. But a joint estimator is sensitive to this prior joint distribution and to the relative uncertainty placed on the initial parameters and kinematics. This relative uncertainty is dictated by the relative noise covariances in the prior models of kinematics and parameters in Eqs (1) and (8), and by the selected covariances on their initial estimates. For example, if parameters are initially poor but not enough noise is placed on their prior model, the joint estimator will likely not converge as it assumes that the parameters are closer to the true values than they actually are; hence it will make most of the update to the decoded kinematics while assuming the wrong parameters. This worsens the estimate of the kinematic intention, which in turn further disrupts the parameter estimation. In contrast, adaptive OFC-PPF convergence does not depend on the initial kinematic decoder quality; the only way the decoded kinematics enter the adaptation process is by providing the visual feedback term in the OFC model since the monkey indeed observes these decoded kinematics regardless of their quality.

It is also important to note that in our architecture the estimation of each neuron’s parameters is independent of other neurons and only requires the OFC-inferred intention. Hence the parameter decoders for each neuron can run in parallel if need be, making it readily scalable to many channels of recording. Moreover, the state dimension is relatively small (3 in this work) for each parameter decoder and hence the estimation is not computationally demanding. For example, our system could be run in experiments with 20–30 neurons and 5ms bins within MATLAB even using a serial implementation of parameter decoders.

#### Autonomous and dynamic assisted training using the OFC-PPF paradigm

To enable the BMI to perform general movements (e.g., curved jumps or straight target-directed movements), once decoder parameters converge we use a random-walk prior model (c.f. Eq (13) with **L**_*a*_ = 0 and Eq (19)) in the kinematics decoder. This random-walk prior model only enforces two general rules, which apply to many types of movements, without any additional assumptions: 1) velocity evolution in time is correlated, i.e., there is correlation between velocity vectors at two adjacent time points; 2) position is the integral of velocity. Given the poor initial parameter estimates, however, an initial random-walk kinematic decoder often generates trajectories that are far from the intended trajectories. This may result in decoded trajectories that are biased towards a specific region of space, and consequently cause slower convergence in decoder parameters by not allowing the decoder to explore the space. Moreover, this may result in low initial success rates and hence in reduced subject engagement in the task, which can consequently lead to failure in parameter convergence.

Here we develop a new dynamic assisted training technique within the OFC-PPF paradigm that allows the subject to explore the space even with poor initial parameters, and hence results in consistent parameter convergence across sessions. Assisted training has been used in prior studies to help subjects reach targets with a poor decoder (e.g., [6, 11, 36]). Current methods either add to the decoded velocity vector an assistive vector that directs straight towards the target, or subtract from the decoded velocity vector a vector perpendicular to the straight line to the target, thus attenuating the deviation. These methods often continuously or manually change the ratio of the assistive vector to the decoded vector in the sum to adjust how much assistance is provided to the subject. These ad-hoc approaches can make convergence trainer-dependent and significantly reduce robustness. Highly variable levels of assistance can also complicate the interpretation of performance during adaptation. It is, therefore, important to develop an assisted training technique that provides a systematic way to change the level of assistance before parameter convergence, and also enables a way to compute a chance level assisted performance, the comparison to which may provide a potential approach to assess user’s engagement during assistance. Moreover, while current methods often set a constant speed for the assistive vector, it is important to provide an approach to adjust the prosthetic speed systematically in a given trial. Finally, it could be beneficial to incorporate more naturalistic modulations of speed compared to current manipulations (such as adding to the decoded vector), which for example do not enforce a stopping condition at the target.

Our new OFC-based assisted training technique automatically and dynamically adjusts the assistance level based on subject’s performance, and can provide a way to calculate a chance level performance during assisted training; this may in turn have the potential to assess subject engagement during assistance. The assist technique automatically iterates between assist and test periods and stops assistance once proficient performance is reached. The OFC-based assisted training also automatically adjusts the speed at each time step based on the current kinematic feedback. It also enforces stopping at the target. Finally, it can be combined with the OFC-PPF spike-event-based adaptation and intention estimation in a modular fashion and does not interfere with the process of adaptation.

**Fig 2:**
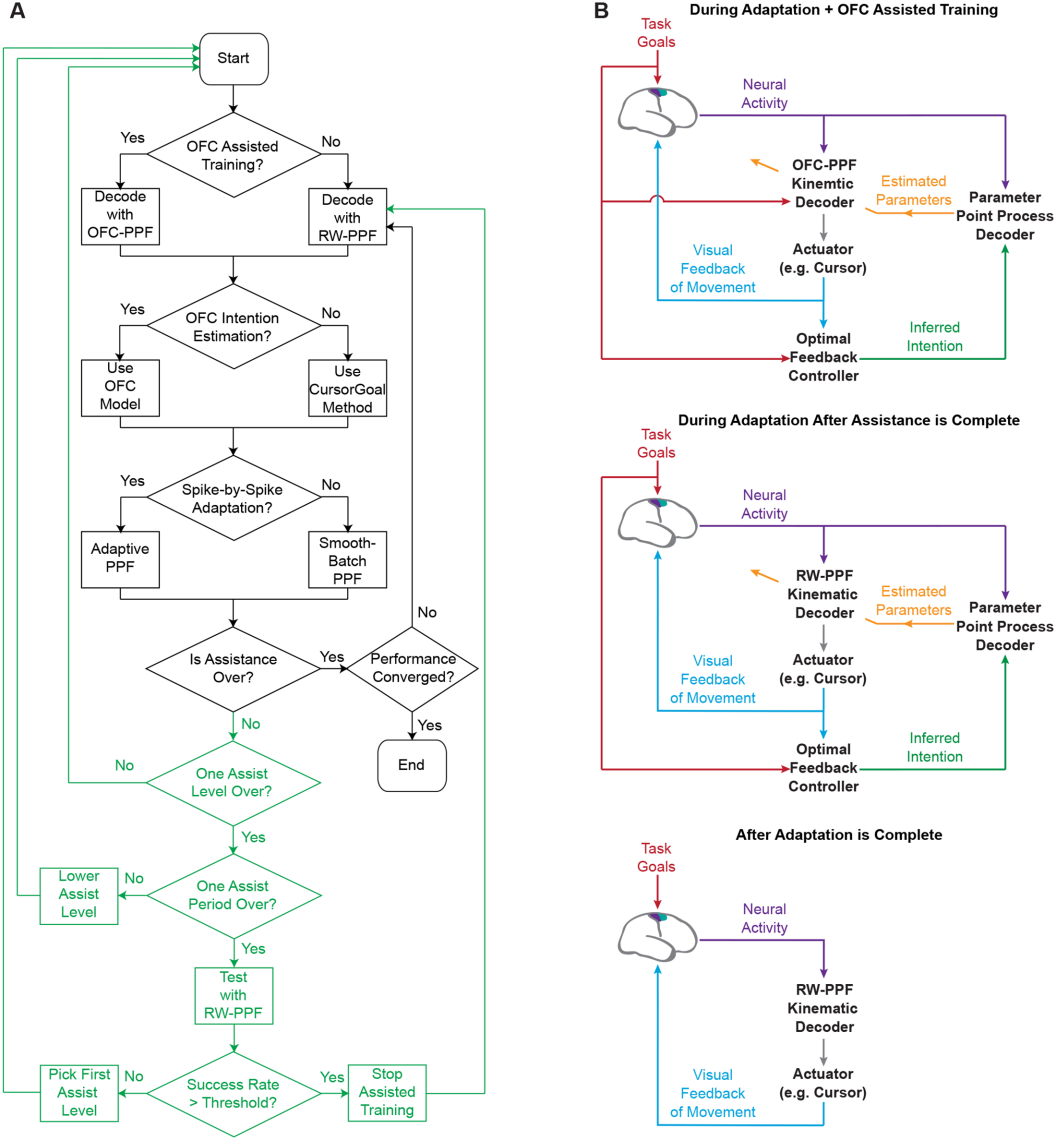
Adaptive OFC-PPF flow-chart. (A) The architecture proceeds with decoding as follows. The decoder parameters are first initialized as desired. The architecture then starts a period of combined assisted training and spike-event-based closed-loop adaptation. Once non-assisted performance in a test period exceeds a desired threshold, assistance stops and a random-walk PPF (termed here RW-PPF) is used for control. Spike-event-based adaptation continues until performance saturates. At that point, adaptation stops and the random-walk PPF with the converged parameters is used by the subject for volitional control of movements in various tasks. The green parts of the flow-chart show the dynamic assisted training procedure. (B) The architecture during the different stages of adaptation and assisted training.

The OFC-based assisted training works by incorporating the target information in the kinematics decoder using an optimal feedback-controlled prior kinematics model instead of the random-walk model (Fig 2). This results in a target-directed decoder that, in addition to enforcing continuity, reflects the subject’s goal to reach the instructed target in the decoded kinematics [26, 39]. Specifically, we build the prior model of our target-directed decoder using Eqs (1) and (3) as

$$\begin{array}{c} x_{t}=\left(A-BL_{a}\right)x_{t-1}+BL_{a}x^{*}+w_{t-1}. \end{array}$$ (13)

Note that **L**_*a*_ depends on the cost function chosen in Eq (2) for assisted training and need not be equal to **L**, which is used for intention estimation. Indeed, to change the level of assistance dynamically, we systematically change **L**_*a*_ depending on the subject’s performance as we describe below. In contrast, **L** for intention estimation remains constant throughout the adaptation process.

Given the prior state-space model in Eq (13) and the observation model in Eq (4), we can find the recursions of the kinematics decoder similar to what we have derived for a feedback-controlled point process decoder for target-directed movements [26, 39]. Let’s denote the one step prediction mean by **x**_*t*|*t*−1_ = *E*(**x**_*t*_|**N**_1: *t*−1_), the prediction covariance by **Λ**_*x*_*t*|*t*−1__, the MMSE estimate that is displayed to the subject by **x**_*t*|*t*_, and its covariance by **Λ**_*x*_*t*|*t*__. Using Eq (13), the prediction step of OFC-PPF for the kinematics is found as

$$\begin{array}{cc} x_{t|t-1} & =\left(A-BL_{a}\right)x_{t-1|t-1}+BL_{a}x^{*} \end{array}$$ (14)

$$\begin{array}{cc} \Lambda_{x_{t|t-1}} & =\left(A-BL_{a}\right)\Lambda_{x_{t-1|t-1}}{\left(A-BL_{a}\right)}^{T}+W \end{array}$$ (15)

The decoder update step for the log-linear rate function in Eq (5) as we have shown in S2 Text and derived previously [26] is given by

$$\Lambda_{x_{t|t}}^{-1}=\Lambda_{x_{t|t-1}}^{-1}+\sum_{c=1}^{C}{\tilde{\alpha }}_{t-1|t-1}^{c}{\tilde{\alpha }}^{c^{T}}{}_{t-1|t-1}\lambda_{c}(t|v_{t|t-1},\phi_{t-1|t-1}^{c})\Delta$$ (16)

$$\begin{array}{cc} x_{t|t} & =x_{t|t-1}+\Lambda_{x_{t|t}}\sum_{c=1}^{C}{\tilde{\alpha }}_{t-1|t-1}^{c}\left(N_{t}^{c}-\lambda_{c}\left(t|v_{t|t-1},\phi_{t-1|t-1}^{c}\right)\Delta \right) \end{array}$$ (17)

where ${\tilde{\alpha }}_{t-1|t-1}^{c}={\left[0,\alpha_{t-1|t-1}^{c}\right]}^{T}$, since the observation model assumes velocity tuning only and no tuning to position (see also [34] for a general treatment). Note that, as before, $\phi_{t-1|t-1}^{c}={\left[\beta_{t-1|t-1}^{c},\alpha_{t-1|t-1}^{c}\right]}^{T}$ are the estimated parameters found in Eq (12). Hence, during assisted training, adaptive OFC-PPF decodes the kinematics using Eqs (14)–(17). In this work we use a special case of the above by implementing a velocity PPF; this means that in the update steps in Eqs (16) and (17), we only update the velocity and set the position equal to the predicted position in Eq (14), which is equal to the integral of the decoded velocity (this is in the same spirit as a velocity KF; see the specific choices of **A** and **B** below).

Similar to the parameter recursions in Eqs (9)–(12), these kinematic recursions are obtained from the same general form given in S2 Text, but this time by taking the parameters as known and decoding the kinematics instead. Since all *C* neurons are informative of the kinematics, the kinematic decoder incorporates all neurons’ spiking activities. For *C* neurons and assuming that their activities are independent conditioned on the kinematic state, the correction term simply becomes the sum of the correction terms for each neuron. In the prediction step, we use the prior model of kinematics in Eq (13) (either assisted or non-assisted) to predict the kinematics at time *t* based on the estimated kinematics at time *t*−1. In the update step, we correct this prediction based on the difference between the spike observations of all neurons $N_{t}^{1:C}$ and their predicted probability of having a spike at time *t*, i.e., $\lambda_{c}\left(t|v_{t|t-1},\phi_{t-1|t-1}^{c}\right)\Delta$. This is the predicted probability since the predicted kinematics **v**_*t*|*t*−1_ is used to compute it. Note that here we consider $\phi_{t-1|t-1}^{c}$ as given and decode the kinematics. The more informative the spiking activity is about the kinematics, i.e., the larger $\phi_{t-1|t-1}^{c}$ is for a neuron, the more weight is placed on the correction term for that neuron. If the spiking activity is not informative at all for a neuron, or $\phi_{t-1|t-1}^{c}=0$, then that neuron’s spiking activity will not affect the decoded kinematics (see also S3 Text).

The assisted training paradigm can dynamically change the level of assistance. To decrease the level of assistance, we change **L**_*a*_ by increasing *w*_*v*_ and *w*_*r*_ in the cost function in Eq (2). Increasing these weights increases the time it takes for the state-space model in Eq (13) to reach the target. A longer arrival time at the target is equivalent to a lower level of assistance since the subject would need to take the cursor to the target by relying more on its neural activity as opposed to the prior model (c.f., Eqs (14) and (17). Motivated by [44], we pick

$$\begin{array}{c} w_{v}=1.5\tau^{2},\;w_{r}=\tau^{4}, \end{array}$$ (18)

where *τ* is a time constant. Hence we change the level of assistance by changing this single parameter *τ*. As *τ* → ∞, **L**_*a*_ → **0**, the state-space model in Eq (13) becomes equivalent to a random-walk state-space model, and hence assistance stops.

The dynamic assisted training paradigm proceeds as follows (Fig 2). We first decide on a discrete set of assistance levels corresponding to a discrete set of *τ*’s, and on a time period to decode using each level of assisted training. We start the adaptive OFC-PPF from the highest assist level and decrease the assist level one by one while the subject is performing the task and parameters are being updated. After the time period for the lowest assist level ends, we initiate a test period in which the subject controls the BMI with no assistance, i.e., using a random-walk state-space model in Eq (13) corresponding to *τ* → ∞. We evaluate the subject’s performance during this test period. Based on this non-assisted level of performance, the system decides on whether assistance should continue and if so, what level of assistance it should restart at. The process enters a new assist period and repeats until non-assisted performance exceeds a desired threshold. Note that parameter adaptation continues even after assistance stops. Indeed we can set the performance threshold, which is used to decide whether to continue the assistance, below the maximum steady-state performance that happens once parameters fully converge.

#### Chance level performance computation during assisted training

**Fig 3:**
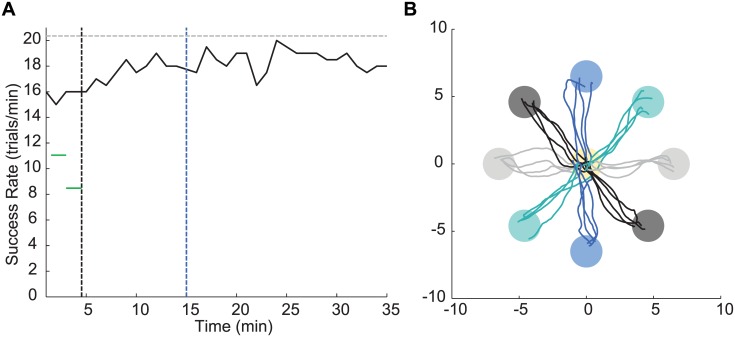
Performance over the process of adaptive OFC-PPF. (A) Success rate as a function of time from the start of the experiment in one session. Success rate is calculated in sliding 2 min windows. The decoder was initialized using a visual feedback seed. Adaptive OFC-PPF was then run as described in the flow-chart in Fig 2. The architecture started by providing assisted training and adaptation. After the first assist period, which consisted of 3 discrete assist levels, performance in the test period exceeded the desired threshold of 5 trials/min and hence the architecture stopped the assisted training (vertical dashed black line). We stopped the adaptation at the vertical dashed blue line, after which the trained point process model was used in a random-walk PPF to control the cursor. Green lines show the 99% upper bound on the chance level performance during the assisted training. Assisted performance is above the 99% chance level. The horizontal dashed line shows the mean manual task performance with the arm on that day. (B) Randomly selected center-out trials on this day after adaptation stopped.

While providing assisted training, it is often difficult to assess whether subjects are engaged in the task. This is because a high level of assist could result in good performance even in the absence of volitional control by the subject. In addition to providing an automatic way to conduct dynamic assisted training, our architecture provides a way to compute the chance level performance during assisted training. The ability to compute this chance level may have the potential to assess subject engagement during assisted training in a principled manner. We calculate the chance level performance at each assistance level by running the adaptive OFC-PPF while the subject’s monitor is off and hence the subject cannot be engaged in the task. In this case, the adaptive OFC-PPF performance is purely due to the assisted state-space model. If a subject’s performance is significantly larger than the corresponding chance level performance during a given assist level, this may indicate that the subject is engaged in the task. This could in turn be useful both for identifying assist levels that enable subject engagement and for post-hoc interpretation of performance during assistance. We have shown the chance level performance with assistance in green in Fig 3. We can see that performance with assistance is above the 99% chance level; consistent with this, the monkey’s performance converges. In this monkey, assisted training always allowed it to converge to a good decoder. We did not observe instances of the monkey giving up with assisted training (in contrast to without assisted training). Had we observed sessions in which the decoder did not converge, it would have been interesting to confirm that the assisted performance would fall below chance level in such sessions. While in principle this computational approach to find the chance level assisted performance may allow us to assess engagement, we need additional experiments with a less motivated animal to fully test the idea. This will be the focus of our future investigations.

#### State-space model for 2D cursor movements

While the OFC-PPF development above is general to any state-space model, in this work we pick the forward dynamics model in Eq (1) as

$$\begin{array}{cc} & A=\left[\begin{array}{cccc} 1 & 0 & \Delta & 0\\ 0 & 1 & 0 & \Delta \\ 0 & 0 & a & 0\\ 0 & 0 & 0 & a \end{array}\right] \end{array}$$ (19)

$$\begin{array}{cc} & B=\left[\begin{array}{cc} 0 & 0\\ 0 & 0\\ \Delta & 0\\ 0 & \Delta \end{array}\right] \end{array}$$ (20)

$$\begin{array}{cc} & W=\text{diag}\left[\begin{array}{cccc} 0 & 0 & w & w \end{array}\right] \end{array}$$ (21)

where *a* and *w* are fit to the monkey’s own end-point cursor kinematics using maximum-likelihood estimation [21]. Moreover, we use a velocity decoder, i.e., we decode the velocity and obtain the position deterministically by integrating the decoded velocity in each dimension using **x**_*t*_ = **Ax**_*t*−1_, i.e., $d_{t|t}^{\text{horiz}}=d_{t-1|t-1}^{\text{horiz}}+\Delta v_{t-1|t-1}^{\text{horiz}}$ and similarly for the vertical position.

#### Adaptive OFC-PPF: A fully modular closed-loop BMI architecture

The complete flow chart for the adaptive OFC-PPF architecture is shown in Fig 2. The architecture proceeds with decoding as follows. The decoder parameters are first initialized as desired. The architecture then starts a period of combined assisted training and spike-event-based closed-loop adaptation. Once non-assisted performance exceeds a desired threshold, assistance stops and a random-walk PPF is used for control. Spike-event-based adaptation continues until performance saturates. At that point, adaptation stops and the random-walk PPF with the converged parameters is used.

It is important to note that the adaptive OFC-PPF architecture is fully modular. First, the intention estimation module is fully independent of the rest of the architecture. For example, one can elect to use prior methods of intention estimation [20] without modifying any other OFC-PPF modules. The assisted training module is also fully separate from the rest of the architecture and its use is optional. In particular, OFC assisted training and intention estimation are separate modules and can use different cost functions and can be used independently. Moreover, the spike-event-based method of adaptation is a module independent of the kinematics decoder and intention estimation. For example, one can use the SmoothBatch method for updating the parameters while keeping the rest of the architecture intact.

### Experimental Procedures

#### Surgery and electrophysiology

A male rhesus macaque (Macaca mulatta) was chronically implanted with arrays of 128 Teflon-coated tungsten microwire electrodes (35 *μm* diameter, 500 *μm* spacing, 8 × 16 configuration; Innovative Neurophysiology, Durham, NC). The arrays targeted the arm and hand areas of the primary motor cortex (M1) and dorsal premotor cortex (PMd) of each hemisphere. Localization of target areas was performed using stereotactic coordinates and implants were typically positioned at a depth of 2.5 *mm*.

Neural activity was recorded using a 128-channel MAP system (Plexon, Inc., Dallas, TX, USA). For real-time BMI control, channel-level activity was used, which was defined by setting thresholds for each channel (5.5 standard deviations from the mean signal amplitude) and using online sorting software to define unit templates that captured all incoming neural activity on the channel. Thresholds were set at the beginning of each session based on 1–2 min of neural activity recorded as the animal sat quietly. After defining channel thresholds, the online sorting client was used to define unit templates. Templates were typically defined that captured all threshold crossings. In the rare event that channel-level activity showed clearly separable units that could not be adequately captured in a single sorting template, the channel was split into two units. The use of templates allowed for real-time rejection of non-neuronal artifacts during BMI sessions. BMI was controlled by 15–20 multi-units on each day. We used 5ms as we found that more than 1 spike rarely occurred within a single 5ms bin (less than 0.6% of the bins).

All procedures were conducted in compliance with the National Institutes of Health Guide for the Care and Use of Laboratory Animals and were approved by the University of California, Berkeley Institutional Animal Care and Use Committee.

#### Decoder initialization

Decoder parameters were initialized either by fitting them using neural activity recorded as the subject passively viewed a cursor moving through the center-out task during a visual feedback session (referred to as visual feedback seed) [21], or they were initialized arbitrarily by randomly permuting the visual feedback seed across neurons.

#### Behavioral tasks

*Center-out task.* The monkey performed a self-paced delayed center-out reaching task under BMI control (Fig 1A and 1B). During BMI control, its arm was confined within a primate chair. Trials were initiated by moving to the center target. Upon entering the center, one of eight peripheral targets appeared. After a brief hold, a “go” cue (center target changing color) indicated to the subject to initiate a reach to the peripheral target. Successful trials required a movement to and brief hold at the central target to initialize a trial, followed by a movement to and brief hold at the peripheral target, and were rewarded by a liquid reward. Failure to acquire the target within a specified window or to hold the cursor within the target for the duration of the hold period resulted in an error, and the trial was repeated. To meet the hold requirement, the monkey had to maintain the cursor within the target for the full hold period upon entering it; this is stricter than some prior tasks [20] since the subject had no opportunity to correct for exiting the target before the completion of the hold period. The monkey had to actively return the cursor to the center to initialize new trials after success or failure. Target directions were presented in a blocked pseudo-randomized order. Targets were circular with 1.2 cm radius and were uniformly distributed about a circle of 13 cm diameter. Hold time requirements at the center and at the targets were 250 ms and the subject had 3–10 s to complete the movement from the central to the peripheral target.

*Target-jump task.* The monkey also performed a target-jump task that consisted of the normal center-out trials interleaved with jump trials. In one type of target-jump task, a jump trial occurred with a probability of 25% and in it the target changed randomly to one of the other 7 targets 500 ms after the cursor left the center. In the other type of target-jump task, a jump trial occurred with a probability of 17% and could be one of 5 jump types (displayed later in Figures in Results); again the jump occurred 500 ms after the cursor left the center.

*Target-to-target task.* The monkey also performed a target-to-target movement task in one session. This task required the monkey to perform a point-to-point movement similar to the center-out task. However, this time, there was no center target and the initial and end target locations could be different from the center-out task (displayed later in Figures in Results).

#### Behavioral metrics

BMI performance was quantified using both task-level and kinematic metrics. The main task metric used was success rate (number of successful trials completed per minute), which takes into account both speed and accuracy. We also calculated the trial percent correct (percentage of initiated trials resulting in success). Steady-state success rate was calculated over the best ten minutes of a session and steady-state percent correct was calculated over the best 100 trials per session for all decoders to minimize the effect of motivation variation. In addition to steady-state success rate, we also assess decoder adaptation via the success rate evolution over time by calculating it in non-overlapping 2 min windows. Movement kinematics were quantified using movement error and reach time metrics. Movement error for a given trial was defined as the average perpendicular deviation from a straight-line reach between the central and peripheral target. Reach time was calculated as the time from leaving the central target to arriving at the peripheral target. Average reach time and movement error metrics for a session were calculated in the same manner as the percent correct metric.

## Results

We implemented the adaptive OFC-PPF architecture and dissociated the effect of each of the novel modeling components by performing various closed-loop experiments with a non-human primate. The monkey performed a total of 89 days of experiments with the decoder. Fig 3 shows the result of the full process of adaptive OFC-PPF in an example session from initialization, to assisted training and adaptation, and finally to steady-state control (see also S1 Fig for performance over time after assisted training stops). This figure also shows randomly selected BMI trajectories after adaptation stops and the trained random-walk PPF is used for control by the monkey. In our experiments, we evaluated the adaptive OFC-PPF’s novel CLDA components. In particular, we evaluated the OFC model for intention estimation, the new assisted training algorithm, the spike-event-based method of adaptation, and the extendibility of the architecture to the target-jump and target-to-target tasks.

### Selecting the OFC Model for Intention Estimation

The OFC intention estimation model that we have proposed here can be adjusted by changing the dynamic state-space model in Eq (1) and the cost function in Eq (2). For any prosthetic device with a given dynamic state-space model, once the task goal is chosen, OFC can be used for intention estimation. This may thus provide a general method for intention estimation in different BMI setups. Here for 2D cursor movements, we tried two candidate classes of OFC models for intention estimation. First, we tried a naturalistic OFC model (N-OFC) in which *a* in Eq (19) was fit to the monkey’s own naturalistic cursor movement and in which the cost function was defined as in Eqs (2) and (18). Second, we tried an OFC model, termed instant-OFC, that assumed the monkey’s control command directly sets the intended velocity, i.e., that *a* = 0 in Eq (19). Consequently, in the latter model we set *ω*_*v*_ = 0 in Eq (18) since in this case *u*_*t*_ is equivalent to the intended velocity and hence a non-zero *w*_*r*_ is sufficient to both enforce the stopping condition and impose the control cost.

**Fig 4:**
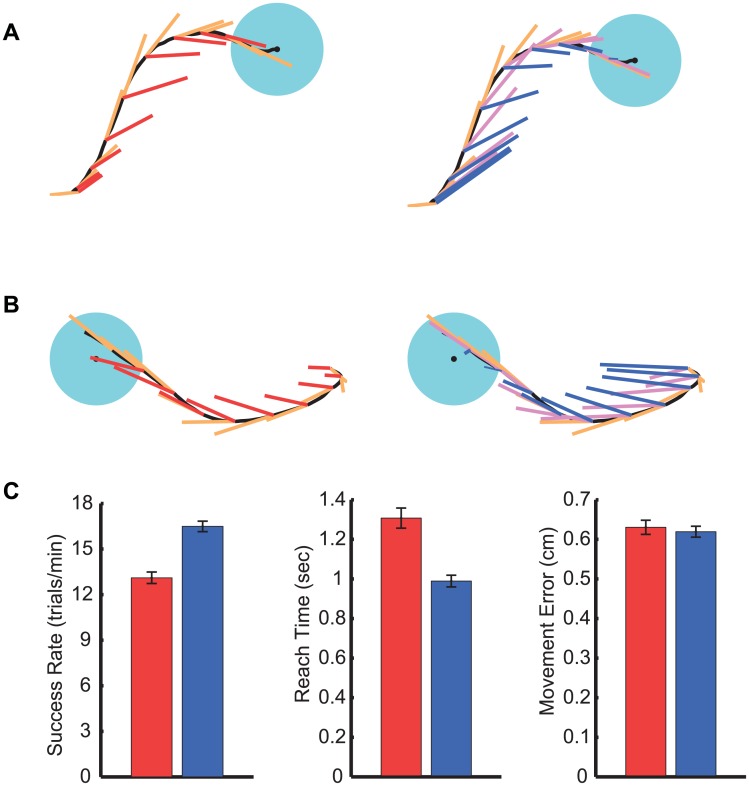
OFC intention estimation results in higher PPF performance. (A, B) Sample decoded trajectories (black), the decoded velocities (orange), and the inferred intended velocities by the N-OFC model (magenta) and the instant-OFC model (blue) on the *right* and by the existing CursorGoal method of intention estimation (red) [20] on the *left*. The latter method [20] obtains the intended velocity by rotating the decoded velocity vectors towards the target while keeping their speed unchanged (the speed is set to zero at the target). Since the instant-OFC model outperformed the N-OFC model, we used the former for intention estimation. (C) Steady-state performance of the PPF decoder trained using the OFC method of intention estimation (blue) vs. the CursorGoal method of intention estimation (red). Bars indicate average values and error bars indicate s.e.m..

Fig 4A and 4B show two sample decoded trajectories, the decoded velocities, and the inferred intended velocities by the N-OFC and the instant-OFC models. The N-OFC model’s inferred intended velocity direction and speed depend on the feedback of both the decoded position and the decoded velocities. The assumption in the N-OFC model is that the monkey intends to gradually change the velocity vector towards the target as opposed to immediately. This is enforced by the non-zero *a* that models the correlation between two adjacent velocity vectors in time. In contrast to N-OFC, the instant-OFC model assumes that the intended velocity need not be correlated with the current velocity (*a* = 0). Hence both its intended velocity direction and speed are mainly dependent on the feedback of the decoded position.

We ran experiments over a total of 18 sessions in which a PPF decoder was trained using either the N-OFC model or the instant-OFC model. We compared the steady-state performance of these two PPF decoders. We found that for 2D cursor movements, using the instant-OFC model compared with the N-OFC model resulted in a PPF decoder that improved success rate by 21% (one-sided *t*-test, *P* < 10^−4^). Movement error and reach times using the instant-OFC model also improved by 6% (one-sided *t*-test, *P* < 0.09) and 15% (one-sided *t*-test, *P* < 10^−3^), respectively (S1 Table, S2 Fig). We also found that the improvement in performance for the instant-OFC model was not due to a simple increase in speed; on average the maximum speed when the decoder was trained with the N-OFC model did not change significantly compared to when it was trained using the instant-OFC model (two-sided *t*-test, *P* = 0.55). This may suggest that the instant-OFC model better captures the user’s control strategy. We thus elected to use the instant-OFC model for intention estimation in this study. Hence OFC-PPF in the remainder of this paper refers to a PPF that was trained with the instant-OFC model.

### OFC Intention Estimation Improves the Performance of the PPF Decoder

To examine the effect of using the OFC intention estimation on the steady-state performance of the PPF decoder, we performed experiments that dissociated its effect and compared it to the state-of-the-art method of intention estimation, i.e., CursorGoal [20]. In particular, in addition to running experiments in which we converged the PPF decoder with the adaptive OFC-PPF, we also ran experiments in which we converged the PPF with exactly the same adaptive approach except for using the CursorGoal method of intention estimation in place of OFC (Fig 2). The CursorGoal method infers the intended velocity direction by rotating the decoded velocity vector towards the target, while setting the intended speed equal to the decoded speed before reaching the target and to zero at the target. We call the PPF decoder trained using the CursorGoal method of intention estimation, CursorGoal-PPF. Fig 4A and 4B show the inferred velocity intentions used in the CursorGoal-PPF for two sample decoded trajectories. These inferred velocities are directed straight towards the target; thus the inferred velocity direction in this case depends only on the current position. Moreover, this method does not infer the speed intention and sets it equal to the decoded speed. Hence a major difference between the instant-OFC model we elected to use and the existing CursorGoal method of intention estimation [20] is that the former infers the speed intention in contrast to the latter that does not infer the speed intention.

We compared the steady-state performance between 12 days with OFC-PPF and 12 days with CursorGoal-PPF (Fig 4C). We found that using the OFC intention estimation significantly improved the performance of PPF decoder (S1 Table), increasing success rate by 26% (one-sided *t*-test, *P* < 10^−6^). Also, reach times were 24% shorter with OFC-PPF compared with CursorGoal-PPF (one-sided *t*-test, *P* < 10^−5^), and maximum speeds with OFC-PPF were higher by 6% compared to CursorGoal-PPF (one-sided *t*-test, *P* < 0.002). Finally, movement error and accuracy did not change significantly between the two decoders (two-sided *t*-test, *P* > 0.6). These results indicate that the monkey could control the OFC-PPF significantly faster than CursorGoal-PPF without a cost on movement error or accuracy. In addition to performance improvement, a principal advantage of using an explicit model of the brain for intention estimation is its ability to extend to prosthetics with various dynamics, simply by incorporating their dynamic model in the OFC formulation (see Discussion).

### OFC Assisted Training Results in Consistent Parameter Convergence

We found that assisted training enabled the monkey to achieve proficient control consistently across days. This was in contrast to days in which assisted training was not applied. Initially during the experiments, we adapted the PPF decoder using the method of SmoothBatch and without the assisted training paradigm. We performed 8 days of experiments in which we initialized the decoder using visual feedback seeds. We found that in 3 of these 8 days, the monkey was unable to converge to proficient control and hence discontinued playing the task. In many cases, the cursor initially got stuck at the boundaries or corners of the workspace and hence was not able to explore the space. In contrast, in tens of days that adaptation was performed with assisted training, whether using the method of SmoothBatch or using continuous spike-event-based adaptation, the monkey always achieved proficient control eventually (see below for comparison of the speed of convergence between SmoothBatch and spike-event-based adaptation). These results suggest that assisted training was essential in keeping the subject engaged and in exploring the space so that decoder parameters could converge.

### Spike-Event-Based Adaptation Results in Faster and More Consistent Performance Convergence

**Fig 5:**
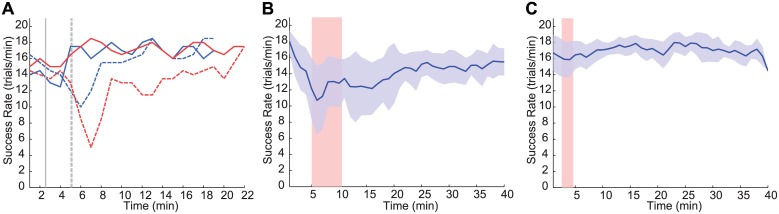
Spike-event-based adaptation enables faster convergence. (A) Performance over time for adaptive OFC-PPF (solid) and SmoothBatch OFC-PPF (dashed) run on two sets (red and blue) of two consecutive days that started from the same initial parameters. Vertical lines show the time point where assistance stopped as the subject’s non-assisted success rate in the test period at that point exceeded the desired minimum threshold of 5 trials/min. Success rate is calculated in sliding 2 min windows. (B, C) Average success rate across sessions as a function of time into the adaptive session for SmoothBatch OFC-PPF in (B) and Adaptive OFC-PPF in (C). Blue curves show the mean success rate over 12 days of experiments for each decoder and shading reflects the standard deviation across these days. The red bar shows the time range in which the BMI architecture stopped the assisted training across days. Spike-event-based adaptation resulted in faster convergence and less variability compared with SmoothBatch adaptation that updated the decoder parameters on a slower adaptation time-scale, i.e., once every 90 seconds.

We investigated whether fast spike-event-based parameter adaptation for the PPF decoder can increase the speed of convergence compared to batch-based methods that update the parameters on the slower time-scale of minutes. To do so, we performed experiments to dissociate the effect of the time-scale of adaptation. In particular, in addition to running experiments in which we trained the PPF using the spike-event-based adaptation method, we also ran experiments in which we trained it with slower adaptation time-scales using the method of SmoothBatch [21], while keeping all the other components of the algorithm the same (Fig 2). SmoothBatch adapts the parameters smoothly once every 90 sec (see Methods). We refer to this decoder as SmoothBatch OFC-PPF. We compared the speed of performance convergence using SmoothBatch OFC-PPF and adaptive OFC-PPF by finding the time it took these decoders to reach 90% of the maximum performance. Fig 5A shows the performance convergence using SmoothBatch OFC-PPF and adaptive OFC-PPF in consecutive days in which parameters were initialized to the same values (see also S3A Fig). Performance converged faster in adaptive OFC-PPF compared to SmoothBatch OFC-PPF in these days. This result held on average across all days as demonstrated in Fig 5B and 5C (see also S2 Table and S3B and S3C Fig). Across 12 days of experiments with each decoder, while the eventual steady-state success rate was the same (two-sided *t*-test; *P* = 0.35), adaptive OFC-PPF converged much faster to this steady-state level compared with SmoothBatch OFC-PPF. In particular, across 12 days, success rate in SmoothBatch OFC-PPF converged in 18.7±3.2 min (mean ± s.e.m.). In comparison and across 12 days, success rate in adaptive OFC-PPF converged in 6.5±0.7 min, which was significantly faster than SmoothBatch OFC-PPF (one-sided *t*-test; *P* < 10^−3^). Hence adaptive OFC-PPF resulted both in faster convergence and in more consistent speed of convergence as indicated by the lower s.e.m. across days. Moreover, assisted training in 2 of the 12 days for SmoothBatch OFC-PPF had to reinitiate after the test period and went on to a second assist period (Fig 2). In comparison, using adaptive OFC-PPF, assisted training always stopped after the first test period since the performance had already exceeded the threshold success rate of 5 trials/min (Fig 5).

### Adaptive OFC-PPF Results in Proficient BMI Control Even from a Poor Initial Decoder

**Fig 6:**
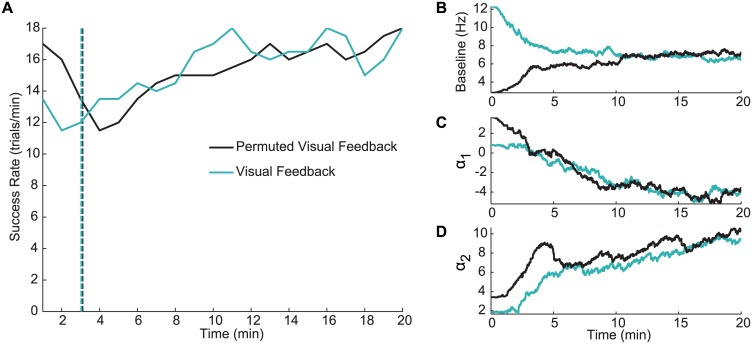
Adaptive OFC-PPF is robust to initialization. (A) Performance over time for adaptive OFC-PPF that was initialized once using a visual feedback seed and once using a permuted visual feedback seed on the same day. Vertical dashed lines show the time point at which the architecture stopped the assisted training. Regardless of the initial seed, performance converges to similar values in these two sessions. Note that initial performance of both visual feedback and permuted visual feedback seeds were poor and hence assistance was used to allow the subject perform the task initially as parameters were adapting. (B–D) Convergence of the point process parameters for an example neuron as a function of time, when starting from the two different seeds. The baseline firing rate is shown in (B) and *α*^*c*^ for the velocity components in the two dimensions are shown in (C) and (D) (see Eq (5)).

We also investigated whether adaptive OFC-PPF could converge to a proficient decoder when starting from a poor initial decoder. First, we found that this monkey was not able to control the BMI using a visual feedback seed decoder (i.e., a decoder whose parameters were found from the neural activity collected during a training session in which cursor movements were visually displayed to the monkey). Success rate using a PPF decoder that used the visual feedback parameters was 0 across all days tried (the monkey soon lost motivation using this decoder, which indeed motivated the design of the assisted training paradigm). Hence across tens of days, adaptive OFC-PPF could always result in proficient performance in this monkey in spite of starting from a poor initial decoder (i.e., the visual feedback seed decoder). To further examine the robustness of adaptive OFC-PPF to initialization, we ran 2 days of experiments in which we started the adaptive OFC-PPF once from a visual feedback seed and once from a seed that was obtained by randomly permuting the visual feedback seed across neurons. We found that adaptive OFC-PPF resulted in proficient control starting from the permuted seed. Moreover, success rate, movement error, percent correct, and reach times did not change significantly between the two converged decoders (two-sided *t*-test; *P* > 0.16; Fig 6A). For example, average success rate for the visual feedback and permuted visual feedback seeds were 14.6 ± 1.8 vs. 14.45 ± 2.3 trials/min, respectively.

We also examined the convergence of decoder parameters starting from the visual feedback and permuted visual feedback seeds using adaptive OFC-PPF (Fig 6B–6D). On the days in which we started adaptive OFC-PPF once from each of these two seeds, we calculated the difference between the estimated parameters in the two cases as a function of time into the adaptive session. We found that this difference was significantly reduced at the end of the adaptive session compared to the start of this session (paired *t*-test; *P* < 0.05). This suggests that the parameters converged to similar values regardless of the initial seed (Fig 6B–6D). In particular, for the baseline firing rate and *α*^*c*^ parameters of each neuron (c.f. Eq (5)), this difference at the start of the session was on average 5.3 and 5.6 times larger than at the end of the session, respectively (see S4 Text for details of calculation).

### Adaptive OFC-PPF Extends to Other Tasks

We also investigated whether adaptive OFC-PPF could extend to tasks beyond the standard center-out target-directed task used for CLDA training. The computational element that allows for this extendability after adaptation is complete is the flexible prior model placed on the kinematics in the PPF (see Eq (13)). After the decoder is trained and assisted training stops (i.e., when **L**_*a*_ = 0), we use a random-walk prior model on kinematics that only constrains the kinematics thorough the dynamic matrix **A** in Eq (19). The **A** matrix just enforces two general rules: 1) velocity evolution in time is correlated, i.e., there is correlation between velocity vectors at two adjacent time points, 2) position is the integral of velocity. These general constraints hold for various types of movement (e.g., straight movements or curved movements). Here we ran closed-loop experiments and showed that the new CLDA technique could enable extension to other tasks.

**Fig 7:**
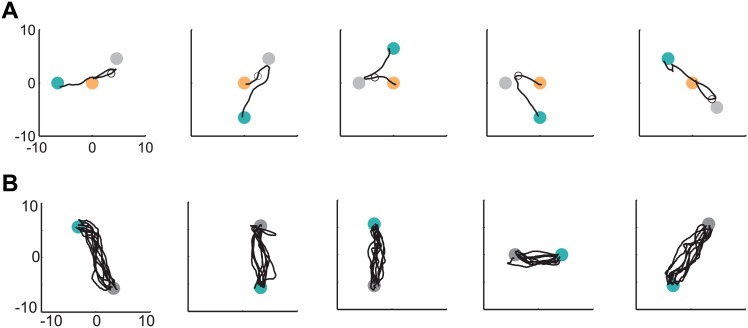
Adaptive OFC-PPF extends to tasks beyond those used for CLDA training. (A) Sample random trajectories in the target-jump task. Gray circle shows the initial target and cyan circle shows the eventual target after the jump occurred. The unfilled circle on the trajectory shows the time at which the jump occurred. The monkey used a random-walk PPF trained on the center-out task to perform this target-jump task. (B) Sample random trajectories in the target-to-target task. Each trial type consisted of a start target and an end target. Instead of going from the center to one of eight peripheral targets in the center-out task, here the monkey had to move the cursor from one target to another target (whose locations could also differ from those in the center-out task).

Using a decoder trained with adaptive OFC-PPF, we had the monkey perform a target-jump task across 7 sessions. In this task, in a random selection of center-out trials, the target jumped to one of the other 7 targets 500ms after the cursor left the center (see Methods; Fig 7A). The monkey achieved proficient control in the target-jump task (Fig 7A). In particular, while the target-jump trials were harder to perform, monkey’s accuracy (percent correct) on these trials was only slightly lower than the normal center-out trials in the same sessions, i.e., 88% vs. 92% (S3 Table). The monkey achieved a success rate of 13.9±0.4 trials per minute on the target-jump task across sessions (note that jumps require moving a longer distance compared to center-out trials; for example, on the same days the monkey’s success rate on the center-out task was 15.08±0.3 in comparison).

In addition to the target-jump sessions, the monkey performed a target-to-target movement task in one session. This task required the monkey to perform a point-to-point movement similar to the center-out task (i.e., movement to and brief hold at an initial target, followed by movement to and brief hold at a terminal target). However, this time, there was no center target and the initial and end target locations could be different from the center-out task (Fig 7B). The monkey again achieved proficient control on this task reaching a success rate of 13.2 trials per minute and an accuracy of 90%.

## Discussion

Significant progress has been made in BMI development by combining Kalman filters with CLDA techniques. However, current decoders such as Kalman filters may limit the time-scale at which the brain can control the prosthetic and the time-scale at which decoder parameters can be adapted. Time-scales of control and adaptation may significantly affect BMI performance. Hence developing decoders that are not limited to a particular time-scale because of modeling assumptions provides a powerful tool to investigate these effects (S1 Text). Moreover, while various successful CLDA approaches have been used in different studies, a unified CLDA framework for intention estimation, assisted training, and adaptation is lacking. Here we propose a novel CLDA architecture for training a PPF BMI in closed-loop operation, which consists of two main computational components: 1. A general unified control-theoretic OFC framework for (i) intention estimation and (ii) assisted training, 2. Dynamic spike-event-based adaptation as opposed to batch-based adaptation. This architecture allows an arbitrarily fast time-scale for spike processing in control and adaptation, and incorporates a general unified control-theoretic CLDA framework. We use the architecture to investigate the effects of the time-scale of adaptation and of the OFC framework on closed-loop decoder training. Using closed-loop experiments in a rhesus monkey over 89 days, we dissociated the effect of the novel components of our CLDA framework, i.e., the OFC method of intention estimation, the OFC method of assisted training, and the spike-event-based time-scale of adaptation in training a PPF. In summary, our paradigm produced improvements on two fronts: 1) spike-event-based adaptation and assisted training improved the speed and ease of decoder convergence during training, and 2) OFC intention estimation resulted in higher performance PPF decoders after training was complete. Overall, the BMI architecture—termed adaptive OFC-PPF—enabled robust performance and extended control to various tasks.

The architecture is based on an infinite-horizon optimal feedback-control model of brain behavior in closed-loop BMI control and a point process model of the spiking activity (Fig 1). The OFC model infers the brain’s intention during the process of adaptation. This OFC model is also used to develop a new assisted training technique, which allows for dynamic and automated assisted training. The assisted training technique also enables the calculation of a chance level performance during assisted training; this may provide a potential approach to evaluate subject engagement during assistance by comparing to this chance level. Thus OFC modeling of brain behavior provides a unified framework for intention estimation and assisted training. The point process model allows subjects to control the BMI with every spike event, and enables the BMI to adapt its parameters with every spike event. The architecture is also fully modular (Fig 2).

Our closed-loop experiments in a non-human primate dissociated the advantage of each of the architecture modeling components. We first dissociated the effect of the OFC intention estimation by comparing OFC-PPF with CursorGoal-PPF at steady state. We found that using the OFC model for intention estimation results in a PPF with higher steady-state success rate compared with current intention estimation techniques (i.e., CursorGoal; Fig 4); this suggests that the former better estimates the brain’s control strategy. We dissociated the effect of the OFC assisted training by comparing OFC-PPF with assisted training turned off or on. We found that the automated assisted training technique results in consistent performance convergence across days by allowing the decoder to explore the space and by keeping the subject motivated. We dissociated the effect of spike-event-based adaptation by comparing SmoothBatch OFC-PPF with adaptive OFC-PPF during decoder training. We showed that fast spike-event-based adaptation results in faster performance convergence compared with existing batch-based methods, which have a slower adaptation time-scale (Fig 5). We also found that the architecture is robust to initialization of parameters (Fig 6). Finally, we showed that adaptive OFC-PPF results in a decoder that extends to tasks beyond those used for CLDA (Fig 3), in particular a target-jump task and a target-to-target task (Fig 7).

While we implemented the OFC model for intention estimation in spike-based BMIs and to fit a point process observation model, the OFC intention estimation technique can also be used for other signal modalities or observation models. These include local field potentials or electrocorticography (ECoG) signals, which can be modeled as a Gaussian process. In these cases, the estimated intention would be used to fit a Gaussian model of neural activity in an appropriate decoder such as a Kalman filter. Moreover, the OFC models the brain’s strategy in closed-loop control of a prosthetic. Since the brain is the controller of movement in any BMI setup, the OFC framework could generalize to various BMI setups by simply quantifying the corresponding prosthetic’s dynamics and the task goals within the OFC’s state-space model and cost function, respectively.

The OFC assisted training technique is also not specific to a point process. We can, for example, extend this framework to provide assisted training in a Kalman filter BMI by using the OFC state-space model as the prior kinematic model in the Kalman filter. The dynamic assisted training will otherwise be identical to the one used in combination with a point process observation model here (Fig 2).

In this work we used a 5ms bin-width to generate the 0 and 1 time-series of the spikes. This was because in our case more than 1 spike rarely occured within a single bin. In the PPF decoder we use a discrete-time point process as an approximation to a continuous-time point process. This approximation is reasonable as long as the bins are small enough to contain at most 1 spike [35]. If in a scenario units or multiunits are recorded with higher firing rates such that there is a considerable probability of having more than 1 spike per 5ms bin, then smaller bin-widths should be used to get a discrete-time point process, which is a good approximation to the continuous-time point process.

An important observation in the BMI field is that the brain can adapt to control a BMI decoder over time [6, 8, 15, 28]. Neural plasticity can result in changes in neural firing properties (e.g., directional tuning) that yield improved BMI performance. Closed-loop decoder adaptation also introduces an additional adaptive element into the BMI, creating a “two-learner system”. Neural and decoder adaptation can interact [28], raising the possibility for interference between these two learning elements. Neural plasticity is most robustly observed with slowly-varying or fixed decoders [15, 28]. A potential concern, then, is that the high frequency of adaptation and control in the OFC-PPF could disrupt neural plasticity. More broadly, how decoder control and adaptation time-scales influence neural plasticity is currently unknown. We therefore devised a set of experiments to examine neural learning effects. We ran an additional experiment with this monkey over 14 days with a semi-stationary neural ensemble using the adaptive OFC-PPF. On each day, adaptation was run for 5–15 minutes starting from the previous day’s decoder. The monkey showed gradual performance improvement over these days, with increased success rate and reduced movement error. These results suggest that learning can indeed occur while the monkey controls a PPF adapted with the spike-event-based adaptation paradigm; thus adaptive OFC-PPF can indeed be combined with brain learning to improve neuroprosthetic control. Detailed data related to the learning experiment are presented elsewhere [28]. The examination of how the time-scales of neural and decoder adaptation may interfere in other scenarios from a theoretical perspective is important in future studies [52].

Another important problem to investigate is the selection of the learning rate in CLDA algorithms. The learning rate is a critical parameter of any adaptive algorithm and is currently selected in an ad-hoc manner and by trial and error in CLDA-based BMIs. The learning rate in adaptive OFC-PPF is dictated by the noise covariance in the state model of the parameter decoder. Developing principled methods for optimal selection of this learning rate will be the topic of our future investigation [50].

The point process model allows subjects to control the BMI and receive feedback at a much faster time-scale than a Gaussian model used in Kalman filters. In this work, we focused on developing the novel closed-loop point process BMI training architecture that is fully modular, adaptive and robust, and can extend control to various tasks. While outside the scope of this work, it is important to investigate whether the PPF can be controlled better than the Kalman filter by using the point process neural encoding model and by providing the fast time-scale of control, which in turn increases the rate of control and feedback. For example, it may indeed be that the faster time-scale of control results in more accurate control. Moreover, the spiking activity that any BMI algorithm uses as its input is fundamentally a sequence of spike events, or zeros and ones, which indicate the time-points at which the spikes occur. The larger bins used in prior decoders just preprocess this spiking signal first, and then use the preprocessed signal (e.g., the count of spikes within the bin) to decode movement intention. As the data processing inequality in information theory states [53], preprocessing data cannot add information. Hence processing the spikes directly with an optimal filter specifically designed for the zero and one spike events could extract the maximal amount of information from the spikes. This, along with the faster update rates, may result in performance improvements. To examine questions related to performance comparisons, novel experiments are needed to dissociate the effects of control rate, feedback rate, and the mathematical encoding model on BMI control. This will be the subject of our future investigation [54]. Finally, in our experiments the smaller time-scale of adaptation resulted in faster convergence to the same steady-state level. However, it would be interesting to examine whether the steady-state performance could also change as a result of the time-scale of decoder adaptation in more complex BMI tasks that rely more on neural adaptation and introduce complex interactions between the decoder and neural adaptation processes.

Here we not only developed the novel adaptive OFC-PPF BMI training architecture, but also dissociated and explored the effect of each of its modeling components. Given the modularity of the architecture and its general control-theoretic CLDA framework, these results guide the incorporation of various proposed modeling components in neuroprosthetic designs. These findings have significant implications towards the development of future clinically-viable neurotechnologies.

## Supporting Information

S1 Text BMI time scales of decoding and adaptation. (PDF)

S2 Text General recursions for a PPF. (PDF)

S3 Text PPF Processing of noisy spiking activity. (PDF)

S4 Text Computing the difference in converged parameters. (PDF)

S1 Fig Performance over the process of adaptive OFC PPF after assisted training. Success rate as a function of time from the start of the session; success rate here is computed and shown only for the portion of the session after assistance stopped. Success rate is calculated in sliding 2 min windows. Figure convention is otherwise the same as in Fig 3A. Note that performance after assisted training ends is above the desired minimum threshold of 5 trials/min.
(TIF)

S2 Fig Instant-OFC method of intention estimation results in more proficient control. Steady-state performance of the PPF decoder trained using the instant-OFC method of intention estimation (blue) vs. the CursorGoal method of intention estimation (red) vs. the N-OFC method of intention estimation (magenta). Bars indicate average values and error bars indicate s.e.m..
(TIF)

S3 Fig Convergence of performance after assisted training. Figure convention is the same as in Fig 5. Success rate is calculated in sliding 2 min windows. Time for each session is aligned to the end of assistance in that session. (A) Non-assisted performance over time for adaptive OFC-PPF (solid) and SmoothBatch OFC-PPF (dashed), which were run on two sets (red and blue) of two consecutive days that started from the same initial parameters. Time is aligned to the end of assisted training for each session. (B, C) Non-assisted average success rate across sessions as a function of time after assisted training stops for SmoothBatch OFC-PPF in (B) and Adaptive OFC-PPF in (C). Time is again aligned to the end of assistance. Blue curves show the mean success rate over 12 days of experiments for each decoder.
(TIF)

S1 Table Performance improvement using instant OFC method of intention estimation. Note that a positive improvement in reach time and movement error means a shorter reach time and a smaller movement error.
(PDF)

S2 Table The effect of adaptation time scale on the speed of performance convergence. (PDF)

S3 Table Percent correct comparison of target-jump vs. center-out trials in the target jump task. (PDF)
