# Supplementary material for: Robust Brain-Machine Interface Design Using Optimal Feedback Control Modeling and Adaptive Point Process Filtering
Source: PLoS Comput Biol. 2016 Apr 1;12(4):e1004730. doi: 10.1371/journal.pcbi.1004730 (PMC4818102; doi:10.1371/journal.pcbi.1004730)
Supplement: S1 Text — (PDF) [file pcbi.1004730.s001.pdf]

S1 Text
Robust Brain-Machine Interface Design Using Optimal Feedback Control Modeling and Adaptive Point Process Filtering

Maryam M. Shanechi[1,2,◑,*], Amy L. Orsborn[3,4,◑], Jose M. Carmena[2–4,*]

1 Department of Electrical Engineering, Viterbi School of Engineering, University of Southern California, Los Angeles, CA, USA
2 Department of Electrical Engineering and Computer Science, University of California, Berkeley, CA, USA
3 Helen Willis Neuroscience Institute, University of California, Berkeley, CA, USA
4 University of California, Berkeley–University of California, San Francisco Graduate Group in Bioengineering
◑These authors contributed equally to this work.
*** shanechi@usc.edu, carmena@eecs.berkeley.edu**

S1 Text: BMI Time-Scales of Decoding and Adaptation

In a CLDA-based BMI architecture there are two important time-scales. First, there is the time-scale of spike processing, decoding, and control, i.e., how often are spike observations used to update the decoded trajectory and hence to generate a new decoded position. In a typical Wiener filter or a typical KF BMI, the inputs to the decoder are spike counts calculated in 50-100ms bins. Hence the time-scale of spike processing and control is 50-100 ms. In contrast, PPF processes every spike event (whether 0 or 1) and hence controls the BMI at a 5ms time-scale. Second, there is the time-scale of adaptation. Batch-based adaptation (e.g., [1, 2]) updates the decoder parameters on the time-scale of minutes. In contrast, spike-event-based adaptation developed here updates the decoder parameters at the 5ms time-scale. We find that spike-event-based adaptation results in faster performance convergence compared with batch-based methods typically used as shown in Fig. 5 in main text.

Note also that the condition of optimality for the Wiener filter or KF is that the spike counts within a bin are approximately Gaussian-distributed. This would require the bin-width (used to count the spikes) to be chosen large enough so that the number of spikes within a bin is large enough to make the count approximately Gaussian-distributed according to the central limit theorem. Hence the bin-width in these decoders could be dependent on whether single-units or multi-units are being recorded in an experiment. For example, for multi-units with high firing rates, the bin-width can be chosen smaller than single-units. Therefore the Wiener filter or the KF constrain the time-scale of processing, control, and adaptation because of their model assumptions. In recent KF studies with multi-units, for example, 50-100ms bins have been used as described above.

References

1. Orsborn AL, Dangi S, Moorman HG, Carmena JM. Closed-Loop Decoder Adaptation on Intermediate Time-Scales Facilitates Rapid BMI Performance Improvements Independent of Decoder Initialization Conditions. IEEE Trans Neural Syst Rehabil Eng. 2012 Jul;20(4):468–477.

2. Gilja V, Nuyujukian P, Chestek CA, Cunningham JP, Yu BM, Fan JM, et al. A High-Performance Neural Prosthesis Enabled by Control Algorithm Design. Nat Neurosci. 2012 Dec;15:1752–1757.
