# Supplementary material for: Robust Brain-Machine Interface Design Using Optimal Feedback Control Modeling and Adaptive Point Process Filtering
Source: PLoS Comput Biol. 2016 Apr 1;12(4):e1004730. doi: 10.1371/journal.pcbi.1004730 (PMC4818102; doi:10.1371/journal.pcbi.1004730)
Supplement: S2 Text — (PDF) [file pcbi.1004730.s002.pdf]

S2 Text
Robust Brain-Machine Interface Design Using Optimal Feedback Control Modeling and Adaptive Point Process Filtering

Maryam M. Shanechi[1,2,◑,*], Amy L. Orsborn[3,4,◑], Jose M. Carmena[2–4,*]

1 Department of Electrical Engineering, Viterbi School of Engineering, University of Southern California, Los Angeles, CA, USA
2 Department of Electrical Engineering and Computer Science, University of California, Berkeley, CA, USA
3 Helen Willis Neuroscience Institute, University of California, Berkeley, CA, USA
4 University of California, Berkeley–University of California, San Francisco Graduate Group in Bioengineering
◑These authors contributed equally to this work.
*** shanechi@usc.edu, carmena@eecs.berkeley.edu**

S2 Text: General Recursions for a PPF

Here we provide the general recursions of the PPF [1] and show how the recursions for the kinematic decoder in (14)–(17) in the main text, and the parameter decoder for each neuron in (9)–(12) in the main text are found as special cases of these recursions. We denote a general state variable (to be decoded) by $\mathbf{h}_t$. For example, the state could be the kinematics or the parameters. We assume that the prior model for $\mathbf{h}_t$ is given by

$$\mathbf{h}_t = \mathbf{G}\mathbf{h}_{t-1} + \mathbf{b} + \mathbf{z}_{t-1} \tag{1}$$

where $\mathbf{z}_t$ is white Gaussian noise with covariance $\mathbf{Z}$ and $\mathbf{b}$ is a constant. We take the observation model as the point process model in (4) in the main text with state $\mathbf{h}_t$, i.e.,

$$p(\mathbf{N}_t|\mathbf{h}_t) = \prod_c \left(\lambda_c(t|\mathbf{h}_t)\Delta\right)^{N_t^c} e^{-\lambda_c(t|\mathbf{h}_t)\Delta} \tag{2}$$

and with a general rate function (i.e., neural tuning) $\lambda_c(t|\mathbf{h}_t)$. Then by making a Gaussian approximation to the posterior distribution at each time [1, 2], the recursions of the PPF are given by:

$$\mathbf{h}_{t|t-1} = \mathbf{G}\mathbf{h}_{t-1|t-1} + \mathbf{b} \tag{3}$$

$$\mathbf{\Lambda}_{\mathbf{h}_{t|t-1}} = \mathbf{G}\mathbf{\Lambda}_{\mathbf{h}_{t-1|t-1}}\mathbf{G}^T + \mathbf{Z} \tag{4}$$

$$\mathbf{\Lambda}^{-1}_{\mathbf{h}_{t|t}} = \mathbf{\Lambda}^{-1}_{\mathbf{h}_{t|t-1}} + \sum_{c=1}^{C}\Bigg[\left(\frac{\partial \log \lambda_c(t|\mathbf{h}_t)}{\partial \mathbf{h}_t}\right)^T \left(\frac{\partial \log \lambda_c(t|\mathbf{h}_t)}{\partial \mathbf{h}_t}\right)\lambda_c(t|\mathbf{h}_t)\Delta - (N_t^c - \lambda_c(t|\mathbf{h}_t)\Delta)\frac{\partial^2 \log \lambda_c(t|\mathbf{h}_t)}{\partial \mathbf{h}_t \partial \mathbf{h}_t^T}\Bigg]_{\mathbf{h}_{t|t-1}} \tag{5}$$

$$\mathbf{h}_{t|t} = \mathbf{h}_{t|t-1} + \mathbf{\Lambda}_{\mathbf{h}_{t|t}} \sum_{c=1}^{C}\left[\left(\frac{\partial \log \lambda_c(t|\mathbf{h}_t)}{\partial \mathbf{h}_t}\right)^T (N_t^c - \lambda_c(t|\mathbf{h}_t)\Delta)\right]_{\mathbf{h}_{t|t-1}} \tag{6}$$

where as in our convention before, $\mathbf{h}_{t|t-1}$ and $\mathbf{\Lambda}_{\mathbf{h}_{t|t-1}}$ are the prediction mean and covariance, respectively, and $\mathbf{h}_{t|t}$ and $\mathbf{\Lambda}_{\mathbf{h}_{t|t}}$ are the posterior mean and covariance, respectively. These recursions are obtained

using (1) for the prediction step and a Gaussian approximation for the update step. This is for a general rate or neural tuning function. If the rate function is log-linear as in (5) in the main text, the second-order derivative vanishes $\frac{\partial^2 \log \lambda_c}{\partial \mathbf{h}_t \partial \mathbf{h}_t^T} = \mathbf{0}$. Hence for the parameter decoder for each neuron c with states taken as $\mathbf{h}_t = \boldsymbol{\phi}_t^c$, $\frac{\partial \log \lambda_c}{\partial \mathbf{h}_t} = \mathbf{s}_t^T$, $\mathbf{G} = \mathbf{I}$, $\mathbf{b} = \mathbf{0}$, $\mathbf{Z} = \mathbf{Q}$, and we get the recursions for each neuron as in (9)–(12) in the main text. For the kinematic decoder with the states taken as $\mathbf{h}_t = \mathbf{x}_t$, $\frac{\partial \log \lambda_c}{\partial \mathbf{h}_t} = \tilde{\boldsymbol{\alpha}}^{c\,T}_{t-1|t-1}$, $\mathbf{G} = (\mathbf{A} - \mathbf{B}\mathbf{L}_a)$, $\mathbf{b} = \mathbf{B}\mathbf{L}_a\mathbf{x}^*$, $\mathbf{Z} = \mathbf{W}$, and we get the recursions in (14)–(17) in the main text. Note that all neurons are encoding the kinematics and we have assumed that neurons are conditionally independent hence we get the summation over all neurons for the kinematic decoder.

References

1. Eden UT, Frank LM, Barbieri R, Solo V, Brown EN. Dynamic analysis of neural encoding by point process adaptive filtering. Neural Comput. 2004;16:971–998.

2. Brown EN, Frank LM, Tang D, Quirk MC, Wilson MA. A statistical paradigm for neural spike train decoding applied to position prediction from ensemble firing patterns of rat hippocampal place cells. J Neurosci. 1998 Sep;18(18):7411–7425.
