# Supplementary material for: Robust Brain-Machine Interface Design Using Optimal Feedback Control Modeling and Adaptive Point Process Filtering
Source: PLoS Comput Biol. 2016 Apr 1;12(4):e1004730. doi: 10.1371/journal.pcbi.1004730 (PMC4818102; doi:10.1371/journal.pcbi.1004730)
Supplement: S3 Text — (PDF) [file pcbi.1004730.s003.pdf]

S3 Text
Robust Brain-Machine Interface Design Using Optimal Feedback Control Modeling and Adaptive Point Process Filtering

Maryam M. Shanechi[1,2,◕,*], Amy L. Orsborn[3,4,◕], Jose M. Carmena[2–4,*]

1 Department of Electrical Engineering, Viterbi School of Engineering, University of Southern California, Los Angeles, CA, USA
2 Department of Electrical Engineering and Computer Science, University of California, Berkeley, CA, USA
3 Helen Willis Neuroscience Institute, University of California, Berkeley, CA, USA
4 University of California, Berkeley–University of California, San Francisco Graduate Group in Bioengineering
◕These authors contributed equally to this work.
*** shanechi@usc.edu, carmena@eecs.berkeley.edu**

S3 Text: PPF Processing of Noisy Spiking Activity

Given that the observations of the PPF are the 0 and 1 time-series and hence noisy, it is interesting to comment on how PPF takes this stochastic noise into account. PPF is a recursive Bayesian estimator. As with any recursive Bayesian estimator, PPF consists of two probabilistic models: a prior model on the kinematic states ((13) in the main text with $\mathbf{L}_a = \mathbf{0}$ after assistance stops) that describes their evolution, and an observation model in (4) in the main text that relates the spiking activity to these kinematic states. At each step of the algorithm, the spike event that is observed (whether 0 or 1) is not the sole determinant of the next kinematic estimate; what determine the next kinematic estimate are the previous kinematic estimate, the prior model, and the spike event. Given a kinematic estimate at time t, the prior model gives us a prediction of the kinematic estimate at time $t + 1$. Then the spike event (0 or 1) makes only a correction to this predication. Hence the prediction step smooths the trajectory. See Materials and Methods for a complete discussion of the PPF recursions in (9)–(12) and (14)–(17) in the main text and how they optimally combine the prior and observation models.
