# Supplementary material for: Robust Brain-Machine Interface Design Using Optimal Feedback Control Modeling and Adaptive Point Process Filtering
Source: PLoS Comput Biol. 2016 Apr 1;12(4):e1004730. doi: 10.1371/journal.pcbi.1004730 (PMC4818102; doi:10.1371/journal.pcbi.1004730)
Supplement: S4 Text — (PDF) [file pcbi.1004730.s004.pdf]

S4 Text
Robust Brain-Machine Interface Design Using Optimal Feedback Control Modeling and Adaptive Point Process Filtering

Maryam M. Shanechi[1,2,◕,*], Amy L. Orsborn[3,4,◕], Jose M. Carmena[2–4,*]

1 Department of Electrical Engineering, Viterbi School of Engineering, University of Southern California, Los Angeles, CA, USA
2 Department of Electrical Engineering and Computer Science, University of California, Berkeley, CA, USA
3 Helen Willis Neuroscience Institute, University of California, Berkeley, CA, USA
4 University of California, Berkeley–University of California, San Francisco Graduate Group in Bioengineering
◕These authors contributed equally to this work.
*** shanechi@usc.edu, carmena@eecs.berkeley.edu**

S4 Text: Computing the Difference in Converged Parameters

We first found the chance level difference between steady-state parameters of a single neuron merely due to the noise in the random-walk model of the parameter decoder. To do so, we calculated the steady-state difference in the same estimated parameter between time samples that were 3.5 minutes apart and hence approximately uncorrelated in time. We then found the difference between the estimated parameters at the start and the end of the session relative to this chance level.
