# Supplementary figures and images for: Robust Brain-Machine Interface Design Using Optimal Feedback Control Modeling and Adaptive Point Process Filtering
Source: PLoS Comput Biol. 2016 Apr 1;12(4):e1004730. doi: 10.1371/journal.pcbi.1004730 (PMC4818102; doi:10.1371/journal.pcbi.1004730)

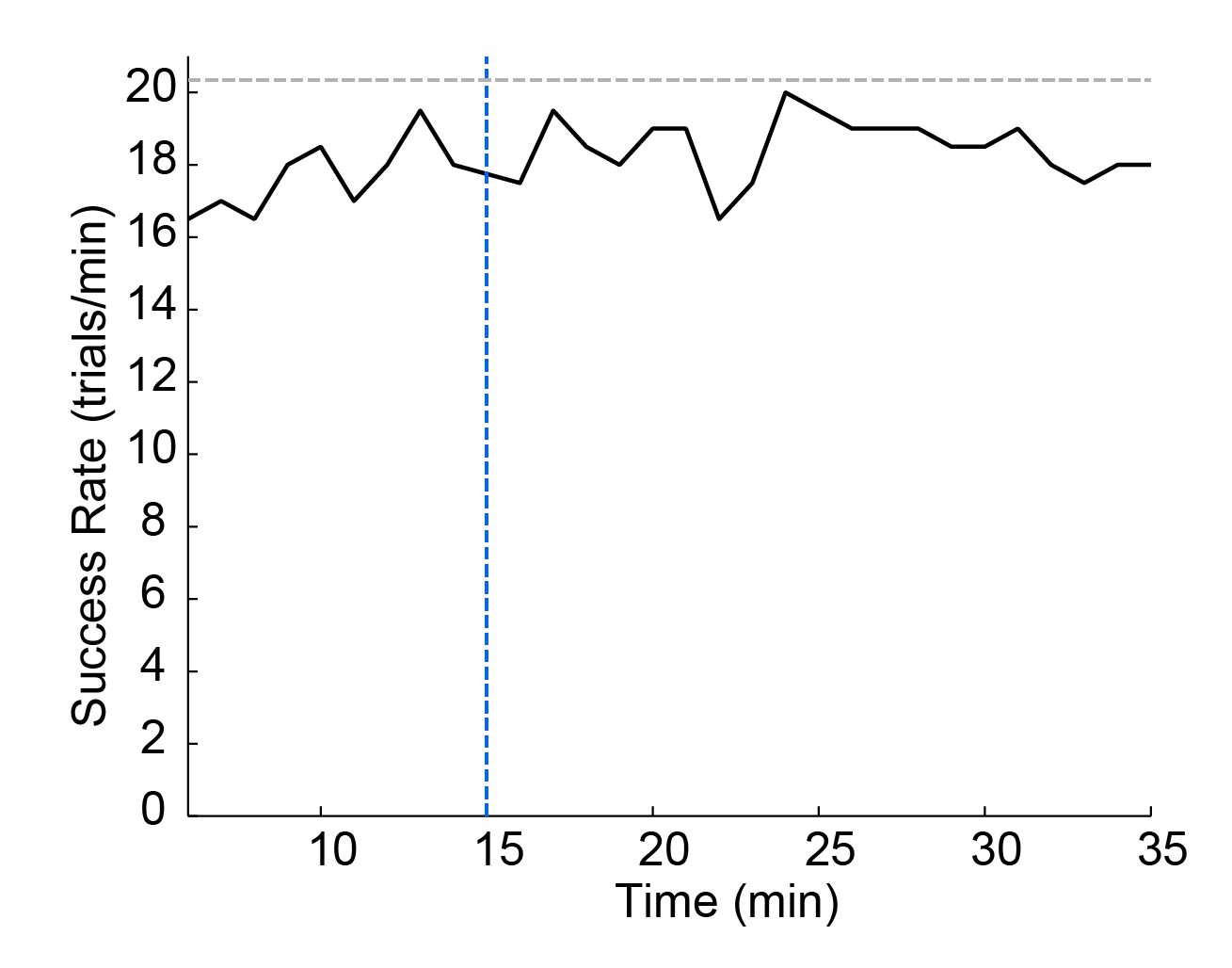

Supplement: S1 Fig — Success rate as a function of time from the start of the session; success rate here is computed and shown only for the portion of the session after assistance stopped. Success rate is calculated in sliding 2 min windows. Figure convention is otherwise the same as in Fig 3A. Note that performance after assisted training ends is above the desired minimum threshold of 5 trials/min. (TIF) [file pcbi.1004730.s005.tif]

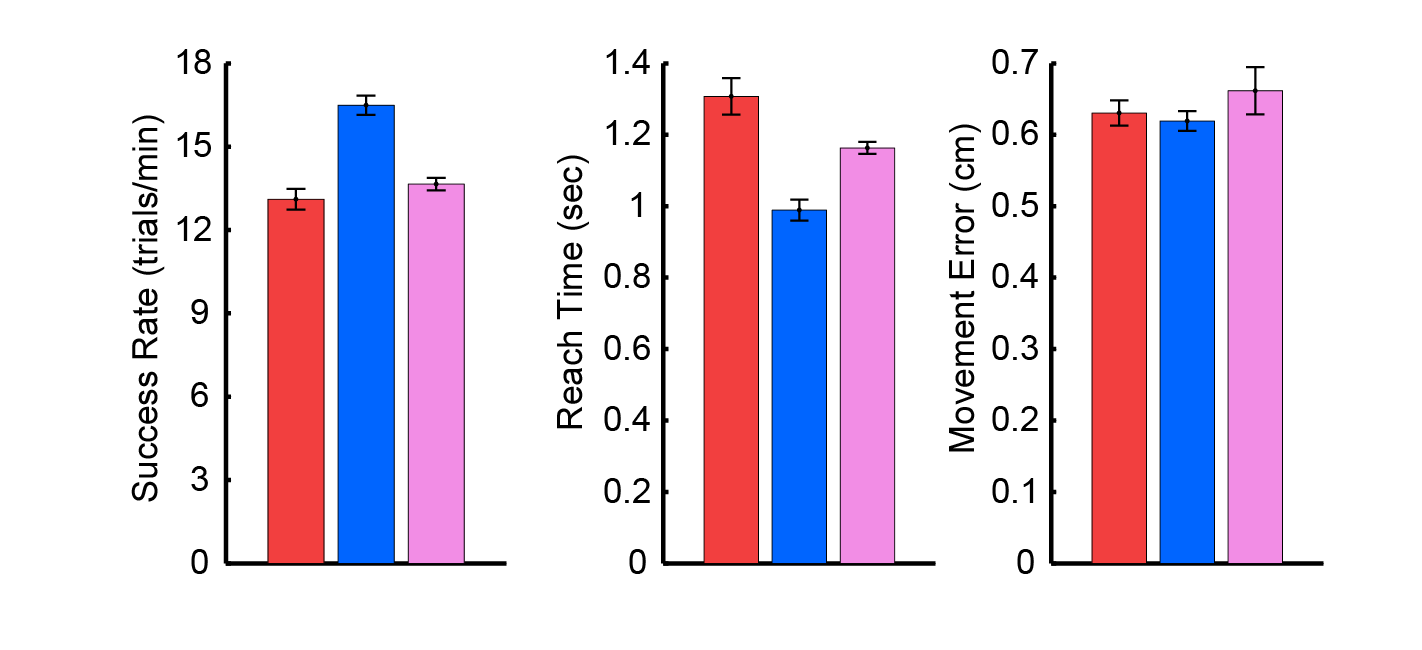

Supplement: S2 Fig — Steady-state performance of the PPF decoder trained using the instant-OFC method of intention estimation (blue) vs. the CursorGoal method of intention estimation (red) vs. the N-OFC method of intention estimation (magenta). Bars indicate average values and error bars indicate s.e.m.. (TIF) [file pcbi.1004730.s006.tif]

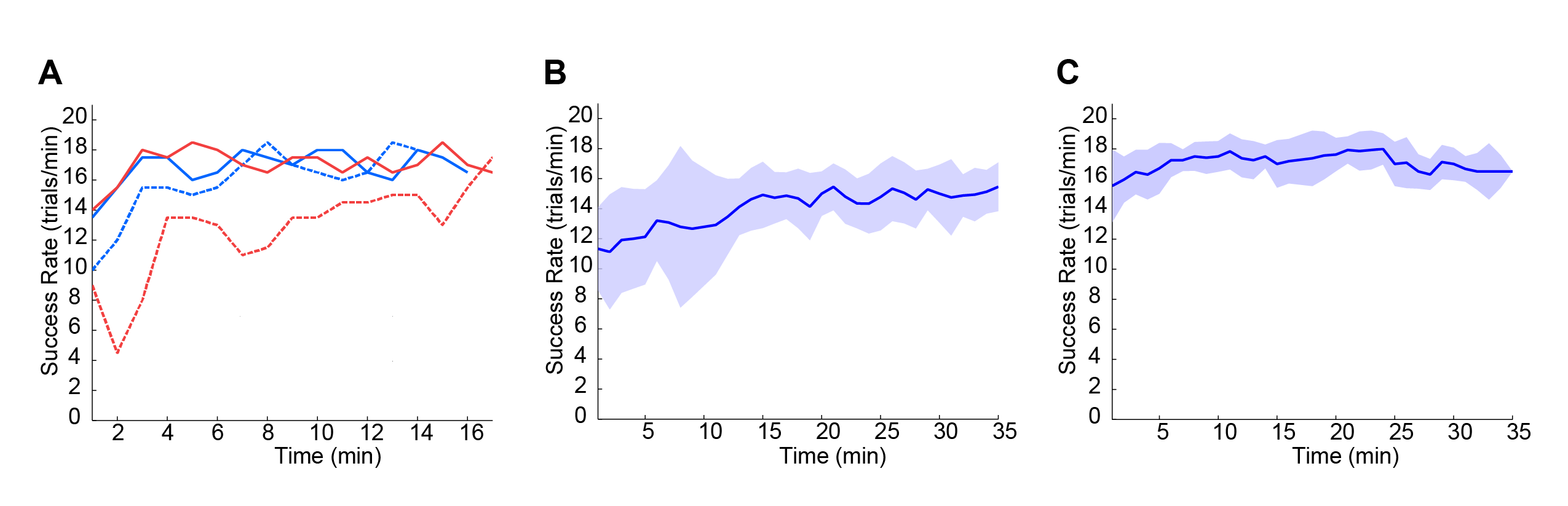

Supplement: S3 Fig — Figure convention is the same as in Fig 5. Success rate is calculated in sliding 2 min windows. Time for each session is aligned to the end of assistance in that session. (A) Non-assisted performance over time for adaptive OFC-PPF (solid) and SmoothBatch OFC-PPF (dashed), which were run on two sets (red and blue) of two consecutive days that started from the same initial parameters. Time is aligned to the end of assisted training for each session. (B, C) Non-assisted average success rate across sessions as a function of time after assisted training stops for SmoothBatch OFC-PPF in (B) and Adaptive OFC-PPF in (C). Time is again aligned to the end of assistance. Blue curves show the mean success rate over 12 days of experiments for each decoder. (TIF) [file pcbi.1004730.s007.tif]
