# Supplementary material for: Robust Brain-Machine Interface Design Using Optimal Feedback Control Modeling and Adaptive Point Process Filtering
Source: PLoS Comput Biol. 2016 Apr 1;12(4):e1004730. doi: 10.1371/journal.pcbi.1004730 (PMC4818102; doi:10.1371/journal.pcbi.1004730)
Supplement: S1 Table — Note that a positive improvement in reach time and movement error means a shorter reach time and a smaller movement error. (PDF) [file pcbi.1004730.s008.pdf]

S1 Table
Robust Brain-Machine Interface Design Using Optimal Feedback Control Modeling and Adaptive Point Process Filtering

Maryam M. Shanechi[1,2,◑,*], Amy L. Orsborn[3,4,◑], Jose M. Carmena[2–4,*]

1 Department of Electrical Engineering, Viterbi School of Engineering, University of Southern California, Los Angeles, CA, USA
2 Department of Electrical Engineering and Computer Science, University of California, Berkeley, CA, USA
3 Helen Willis Neuroscience Institute, University of California, Berkeley, CA, USA
4 University of California, Berkeley–University of California, San Francisco Graduate Group in Bioengineering
◑These authors contributed equally to this work.
*** shanechi@usc.edu, carmena@eecs.berkeley.edu**

S1 Table

Performance Improvement Using Instant-OFC Method of Intention Estimation. Note that a positive improvement in reach time and movement error means a shorter reach time and a smaller movement error.

	instant-OFC vs. N-OFC (%)	instant-OFC vs. CursorGoal (%)
Success Rate	21%	26%
Reach Time	15%	24%
Movement Error	6%	not significantly different
