# Supplementary material for: Robust Brain-Machine Interface Design Using Optimal Feedback Control Modeling and Adaptive Point Process Filtering
Source: PLoS Comput Biol. 2016 Apr 1;12(4):e1004730. doi: 10.1371/journal.pcbi.1004730 (PMC4818102; doi:10.1371/journal.pcbi.1004730)
Supplement: S2 Table — (PDF) [file pcbi.1004730.s009.pdf]

S2 Table
Robust Brain-Machine Interface Design Using Optimal Feedback Control Modeling and Adaptive Point Process Filtering

Maryam M. Shanechi[1,2,◕,*], Amy L. Orsborn[3,4,◕], Jose M. Carmena[2–4,*]

1 Department of Electrical Engineering, Viterbi School of Engineering, University of Southern California, Los Angeles, CA, USA
2 Department of Electrical Engineering and Computer Science, University of California, Berkeley, CA, USA
3 Helen Willis Neuroscience Institute, University of California, Berkeley, CA, USA
4 University of California, Berkeley–University of California, San Francisco Graduate Group in Bioengineering
◕These authors contributed equally to this work.
*** shanechi@usc.edu, carmena@eecs.berkeley.edu**

S2 Table

The Effect of Adaptation Time-Scale on the Speed of Performance Convergence

	Convergence Time (mean ± s.e.m.)
Adaptive OFC-PPF	6.5 ± 0.7 min
SmoothBatch OFC-PPF	18.7 ± 3.2 min
