# Supplementary material for: Robust Brain-Machine Interface Design Using Optimal Feedback Control Modeling and Adaptive Point Process Filtering
Source: PLoS Comput Biol. 2016 Apr 1;12(4):e1004730. doi: 10.1371/journal.pcbi.1004730 (PMC4818102; doi:10.1371/journal.pcbi.1004730)
Supplement: S3 Table — (PDF) [file pcbi.1004730.s010.pdf]

S3 Table
Robust Brain-Machine Interface Design Using Optimal Feedback Control Modeling and Adaptive Point Process Filtering

Maryam M. Shanechi[1,2,◕,*], Amy L. Orsborn[3,4,◕], Jose M. Carmena[2–4,*]

1 Department of Electrical Engineering, Viterbi School of Engineering, University of Southern California, Los Angeles, CA, USA
2 Department of Electrical Engineering and Computer Science, University of California, Berkeley, CA, USA
3 Helen Willis Neuroscience Institute, University of California, Berkeley, CA, USA
4 University of California, Berkeley–University of California, San Francisco Graduate Group in Bioengineering
◕These authors contributed equally to this work.
*** shanechi@usc.edu, carmena@eecs.berkeley.edu**

S3 Table

Percent Correct Comparison of Target-Jump vs. Center-Out Trials in the Target-Jump Task

	Percent Correct
Target-Jump Trials	88%
Center-Out Trials	92%
